# Volatile Fingerprint and Differences in Volatile Compounds of Different Foxtail Millet (*Setaria italica* Beauv.) Varieties

**DOI:** 10.3390/foods12234273

**Published:** 2023-11-27

**Authors:** Miao Kang, Yu Guo, Zhiyuan Ren, Weiwei Ma, Yuewei Luo, Kai Zhao, Xiaowen Wang

**Affiliations:** 1 College of Food Science and Engineering, Shanxi Agricultural University, Jinzhong 030801, China; 18434763724@163.com (M.K.); 18234177373@163.com (Z.R.); a1466173328@163.com (Y.L.);; 2 College of Agriculture, Shanxi Agricultural University, Jinzhong 030801, China; zhaokai017@126.com

**Keywords:** foxtail millet, volatile compounds, electronic nose, volatile fingerprint, HS-GC-IMS, HS-SPME/GC-MS

## Abstract

Aroma components in foxtail millet are one of the key factors in origin traceability and quality control, and they are associated with consumer acceptance and the corresponding processing suitability. However, the volatile differences based on the foxtail millet varieties have not been studied further. The present study was undertaken to develop the characteristic volatile fingerprint and analyze the differences in volatile compounds of 20 foxtail millet varieties by electronic nose (E-Nose), headspace-gas chromatography-ion mobility spectrometry (HS-GC-IMS), and headspace solid-phase microextraction/gas chromatography-mass spectrometry (HS-SPME/GC-MS). A total of 43 volatile compounds were tentatively identified in foxtail millet samples, 34 and 18 by GC-IMS and GC-MS, respectively. Aldehydes, alcohols, and ketones were the major volatile compounds, and the hexanal content was the highest. The characteristic volatile fingerprint of foxtail millet was successfully constructed. A total of 39 common volatile compounds were found in all varieties. The content of hexanal, heptanal, 1-pentanol, acetophenone, 2-heptanone, and nonanal were explored to explain the aroma characteristics among the different varieties, and different varieties can be separated based on these components. The results demonstrate that the combination of E-Nose, GC-IMS, and GC-MS can be a fast and accurate method to identify the general aroma peculiarities of different foxtail millet varieties.

## 1. Introduction

Foxtail millet, which belongs to the gramineous family [1], has been cultivated in China for thousand of years [2]. Foxtail millet is well known for its drought tolerance across the world and is widely planted in arid and semi-arid regions [3]. It is rich in minerals, vitamins, dietary fiber [3], and various biological active compounds, such as catechin, quercetin, and apigenin, with recognized antioxidant, hypoglycemic, antitumor, blood lipid improvement [4], blood pressure reduction, and cancer prevention activities [5], as well as improving the body’s resistance to diseases [6]. With the growing consumer demand for healthy food, the use of foxtail millet as a key ingredient in various millet-based foods, such as porridge, nutrition powders, and steamed bread, has become more prevalent.

Foxtail millet possesses an unique aroma and its major volatile compounds include aldehydes, ketones, alcohols, hydrocarbons, esters, acids, and benzene derivatives [7]. Processing [8,9,10] and storage conditions [11,12,13] greatly affect the aroma of foxtail millet. The concentration of pyrazines and unsaturated aldehydes significantly improves during roasting and boiling, respectively; however, the process of freeze-drying after boiling reduces the contents of volatile compounds and the complexity of foxtail millet porridge [14]. Furthermore, the variety of foxtail millet affects its aroma [7,15]. In China, different varieties of foxtail millet are mainly cultivated in central and northern provinces, such as Shanxi, Inner Mongolia, Shaanxi, Hebei, Henan and Liaoning. To date, the volatile profile and specificity of volatile compounds in different foxtail millet varieties have been sparsely reported, although this information could greatly contribute to a to better understand of processing suitability and origin traceability.

Aroma, as one of the most significant elements impacting the quality and customer acceptability of food, is normally analyzed by electronic nose (E-Nose) [16,17], headspace solid-phase microextraction/gas chromatography-mass spectrometry (HS-SPME/GC-MS) [18,19], and headspace-gas chromatography-ion mobility spectrometry (HS-GC-IMS) [20,21]. E-Nose, which consists of odour sensors and pattern recognition algorithms, is a common intelligent sensing technology that partially eliminates the subjectivity of human sensory evaluation [22,23]. E-Nose is efficient, sensitive, portable, and fast in aroma analysis [24,25,26]. HS-GC-IMS is widely used to analyze the volatile compounds in samples and has the advantages of being fast, sensitive, and easy to pretreat [27,28]. Moreover, HS-SPME/GC-MS can be used to separate volatile compounds from complex samples for qualitative and quantitative analysis [29]. Combining multiple techniques to analyze volatile compounds in samples can result in more comprehensive volatile profiles.

In this study, the volatile compounds in 20 foxtail millet varieties were comprehensively qualitatively and quantitatively analyzed by combining E-Nose, HS-GC-IMS, and HS-SPME/GC-MS, and the characteristic fingerprint of foxtail millet was constructed. Moreover, the similarities and differences in aroma quality of foxtail millet varieties were elucidated by multivariate statistical analysis. The results provide useful information for the identification of foxtail millet and further offer scientific underpinnings for the deep processing development and utilization of the foxtail millet and its products.

## 2. Materials and Methods

### 2.1. Foxtail Millet Samples

Twenty varieties of foxtail millet (Table 1) were planted in the same field under the same environmental conditions and field management in Taigu District, Jinzhong City, Shanxi Province, and then harvested in 2021. All samples were ground into powder after hulling. The resulting flours were stored at −80 °C for further analysis.

### 2.2. Electronic-Nose (E-Nose) Analysis

The volatile compounds in foxtail millet were analyzed using a HERACLES II electronic nose (Alpha MOS, Toulouse, France). For this, 8 g of foxtail millet flour in a 20 mL vial sealed with a magnetic lid was incubated at 40 °C for 30 min. Then, 5 mL headspace was sampled by a syringe and injected into the gas chromatography. Hydrogen was used as the carrier gas and the split flow rate was 10 mL/min at the column heads. Then, the volatile compounds were separated by MXT-5 and MXT-1701 (20 m × 0.18 mm × 0.4 μm, Restek, Co., Bellefonte, PA, USA) column. The initial temperature of the oven was set at 40 °C and held for 10 s, then ramped at 1.5 °C/s until 250 °C and held for 60 s. The temperature of the two flame ionization detectors was set at 250 °C.

### 2.3. Headspace-Gas Chromatography-Ion Mobility Spectrometry (HS-GC-IMS) Analysis

The volatile compounds were analyzed on a GC-IMS (FlavourSpec^®^, G. A. S., Dortmund, Germany). A 20 mL headspace vial with 5 g foxtail millet flour was incubated at 80 °C for 15 min with a 500 rpm agitation rate. A syringe was used to inject 500 μL of headspace gas phase into the injector at 85 °C under splitless mode. The volatile compounds were separated by GC with an MXT-5 capillary column (15 m × 0.53 mm × 1 μm) at 60 °C and coupled with IMS at 45 °C. The carried gas was nitrogen at a programmed flow as follows: 2 mL/min for 0–2 min; 100 mL/min for 2–20 min. The drift gas (nitrogen) was set to 150 mL/min. The retention index (RI) of each volatile compound was calculated using the *n*-ketones C_4_–C_9_ (Sinopharm Chemical Reagent Beijing Co., Ltd., Beijing, China) as external references. Volatile compounds were identified by comparing RI and the drift time with the GC-IMS library.

### 2.4. Headspace Solid-Phase Microextraction/Gas Chromatography-Mass Spectrometry (HS-SPME/GC-MS) Analysis

The volatile compounds in different varieties of foxtail millet were determined by 7610 GC-MS (Thermo Fisher Scientific Inc., Waltham, MA, USA) with a DB-5ms Capillary GC Column (30 m × 0.25 mm × 0.25 μm). For this, 1 g foxtail millet flour was placed into a 20 mL headspace vial with 4 mL saturated sodium chloride and 10 µL of 50 mg/L cyclohexanone as the internal standard, and then sealed with a magnetic lid. The vials with samples were thermostatized at 40 °C for 30 min and then the 50/30 µm DVB/CAR/PDMS SPME fiber (Supelco, Bellefonte, PA, USA) was exposed in the sample vial headspace for 30 min. The fiber was instantly inserted into the injector port at 250 °C for 5 min.

The oven program was set as follows: initial temperature was 35 °C for 2 min; then heated to 280 °C at 5 °C/min and held for 3 min. The inlet temperature was set at 280 °C. The flow rate of helium used as carrier gas was 1 mL/min. The injection mode was splitless. The ion source temperature of mass spectrometry was set at 200 °C with a mass range of 35–400 *m*/*z* and in the electron ionization (EI) mode at 70 eV. *n*-alkanes C_7_–C_30_ (Sigma-Aldrich, St. Louis, MO, USA) were used as external references to calculate the retention index (RI) of volatile compounds. Comparing the mass spectra and RI of volatile compounds in the NIST 11 library, volatile compounds were tentatively identified. The levels of volatile compounds were determined by the internal standard method.

### 2.5. Statistical Analysis

All experiments were repeated three times. Analysis of variance (ANOVA) was performed on the experimental results by BM SPSS Statistics 26 (SPSS Inc., Chicago, IL, USA). MetaboAnalyst was used to conduct the principal component analysis (PCA) for volatile compounds in 20 foxtail millet varieties. Heml 1.0.1 was used to create the heat maps.

## 3. Results and Discussion

### 3.1. Volatile Profile of Foxtail Millet Analyzed by E-Nose

**Figure 1:**
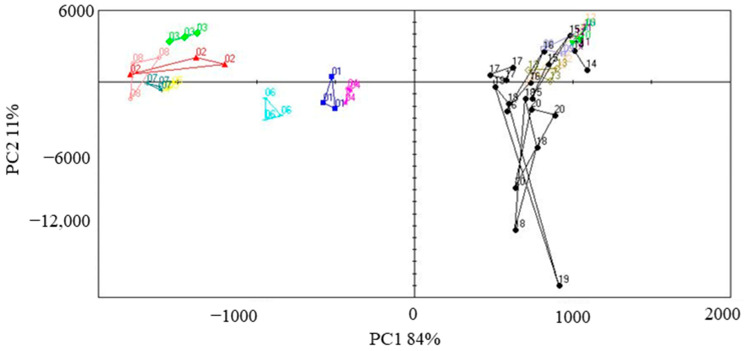
Principal component analysis (PCA) of 20 foxtail millet varieties based on E-Nose.

The volatile profile of foxtail millet was analyzed by E-nose and the results are shown in Figure S1. The peak area of the volatile compound was used to conduct PCA (Figure 1) for understanding the differences in the volatile composition among the foxtail millet varieties. The first two principle components (PCs) accounted for 95% of the total data variability. The 20 varieties of foxtail millet samples were mainly divided into two groups in PC1 (84%): Samples 1 to 8 were placed in the PC1 negative, at the left side of the *x*-axis, while samples 9 to 20 were placed in the PC1 positive, at the right *x*-axis side. Samples 18 (Jingu 62), 19 (Changnong 35), and 20 (Changsheng 07) were grouped in the fourth quadrant, suggesting similar aroma profiles; Samples 1 (77–322) and 4 (Jingu 28) were clustered together and distributed in the third quadrant; Sample 6 (Jingu 34②), located in the third quadrant, was separated with other samples; Samples 2 (Jingu 21), 3 (Jingu 26), 7 (Jingu 34③), and 8 (Jingu 41) gathered together in the second quadrant; other samples were clustered together in the first quadrant. These results showed significant differences in the volatile composition of the 20 foxtail millet varieties, revealing each variety’s specificity.

### 3.2. Volatile Compounds in Foxtail Millet Determined by HS-GC-IMS

**Figure 2:**
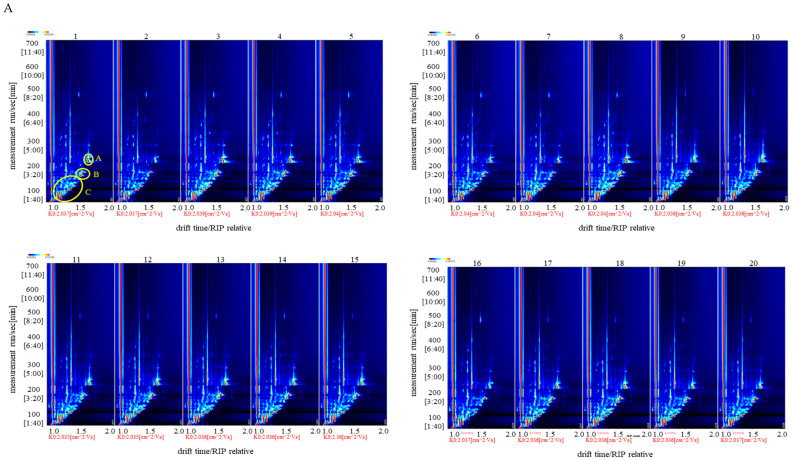

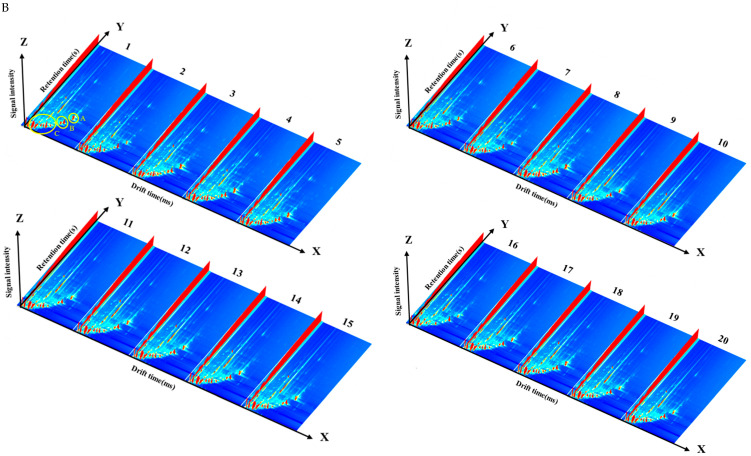

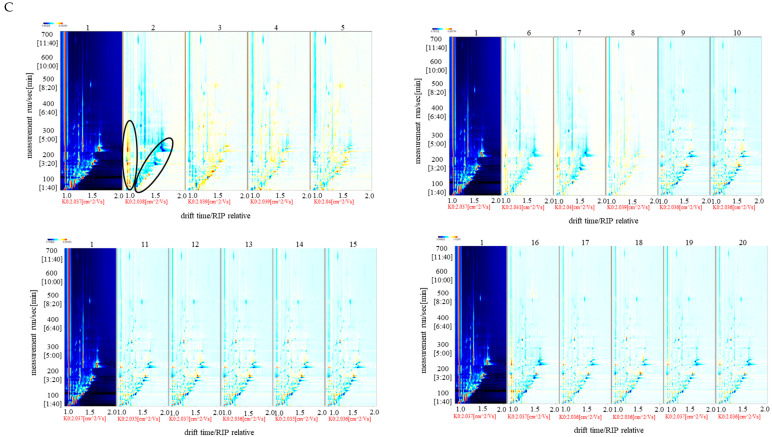
2D topographic photographs (**A**), 3D topographic photographs (**B**), and contrast difference spectra (**C**) of volatile compounds in 20 varieties of foxtail millet.

To analyze the volatile compounds in foxtail millet and discriminate the differences among the 20 varieties, HS-GC-IMS was employed to obtain the global IMS information and identify volatile compounds based on the retention time retention time and ion migration time of gas chromatography [30]. A total of 34 volatile compounds were identified in foxtail millet (Table 2), including 13 aldehydes, 7 alcohols, 6 ketones, 5 esters, 2 furans, and 1 ether. The major volatile compounds in foxtail millet were hexanol, hexanal, 1-pentanol, ethyl acetate and acetone (Figure 2A).

Volatile compounds in foxtail millet were presented in 2D and 3D topographic photographs (Figure 2A,B). Each point on the 2D topographic photographs (Figure 2A) represents a volatile compound, and the color of the point is related to the content of a volatile compound: red for a higher content and white for a lower content [31]. In the 3D topographic photographs (Figure 2B), the transverse, longitudinal and vertical axes represent ion migration time, gas chromatography retention time, and signal peak intensity, respectively. The color and the signal intensity in the vertical axis of the plot are related to the contents of the volatile compounds. As shown in Figure 2A,B, there were many signals dispersed in a region with a GC retention time of 100–200 s and a relative drift time of 1.0–1.5 ms. Based on the fact that non-polar compounds have a longer retention time on non-polar columns than polar compounds [32], it was suggested that volatile compounds in foxtail millet were rich in polar compounds. Although the composition of the volatile compounds resembled those in the various foxtail millet, the content of volatile compounds varied due to variety differentiation (Figure 2A,B). For instance, the ranges of 2-heptanone and hexanol (Area A in Figure 2A,B) in Samples 2 (Jingu 21) and 16 (Jingu 59) were relatively lower than other varieties. The lower contents of hexanal and 1-pentanol (Area B in Figure 2A,B) were observed in Samples 2 (Jingu 21), 6 (Jingu 34②), 7 (Jingu 34③), and 16 (Jingu 59). Also, differences in the components and concentration of volatile compounds were visually found in Area C. To compare the differences in the different foxtail millet more clearly, Sample 1 (77–322) was chosen as the reference, and other varieties were subtracted from the reference on the original basis (Figure 2C). After subtraction, the background color changed to white. A blue plot denotes that the volatile compound’s content is lower than the reference, while a red plot indicates that it is higher than the reference. Differences in the composition and content of volatile compounds were directly observed. These results demonstrate that the volatile compositions of the 20 varieties of foxtail millet were similar, but the volatile contents were significantly different.

**Figure 3:**
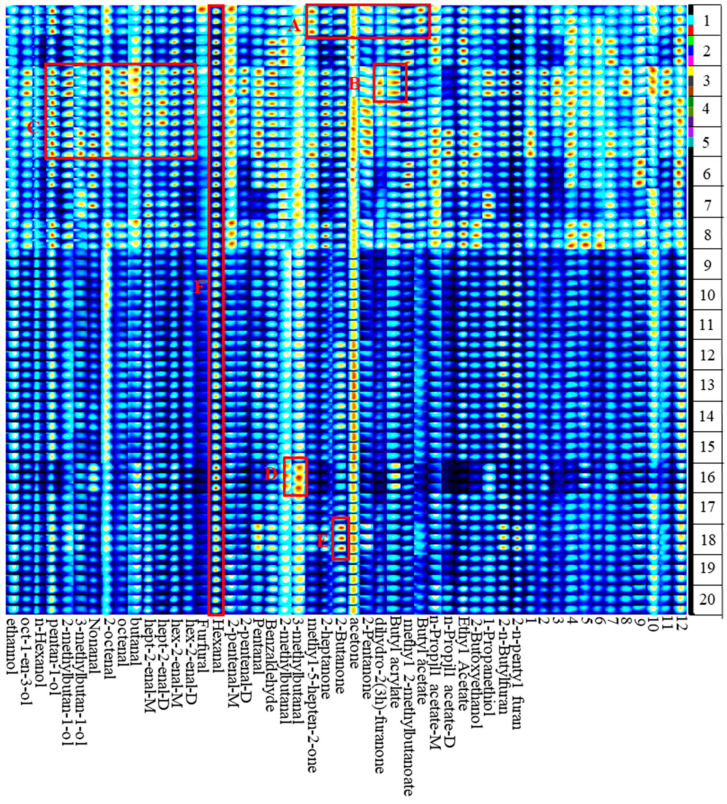
The volatile compounds fingerprints of the 20 foxtail millet varieties.

To reflect the differences in volatile compounds of different foxtail millet varieties, the characteristic non-targeted volatile fingerprints were conducted and analyzed based on the whole spectral information. All the signals were extracted to form the fingerprints (Figure 3), in which each column and row indicated one volatile compound and the sample, respectively. The total signal intensities in Samples 1–8 were obliviously higher than in Samples 9–20, and there were significant differences in volatile compound content between Samples 1–8 and Samples 9–20, which was consistent with the E-Nose results. Methyl-5-hepten-2-one and butyl acrylate reached the maximum concentration in Sample 1 (77–322) (as shown in region A). The intensities of volatile compounds in Sample 3 (Jingu 26) were higher than those in other samples, especially dihydro-2(3H)-furanone and butyl acrylate (as shown in region B). Samples 3 (Jingu 26), 4 (Jingu 28), and 5 (Jingu 34①) in region C had a high content of volatile compounds, and were dominated by alcohols and aldehydes, such as 1-pentanol, 2-octenal, 2-heptenal, and 2-hexenal. The contents of 2-methylbutanal and 3-methylbutanal were highest in Sample 16 (Jingu 59), as shown in region D. The highest content of 2-butanone was found in Sample 18 (Jingu 62) (as shown in region E). Hexanal with strong signal intensity was one of the main volatile compounds in 20 foxtail millet varieties, which is consistent with Liu’s results [32] (as shown in region F).

**Figure 4:**
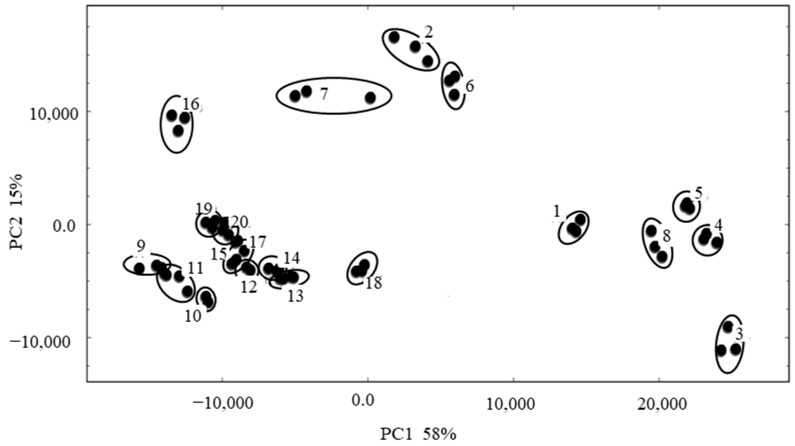
PCA plots of different varieties of foxtail millet.

To better understand the differences in volatile compounds in the 20 foxtail millet varieties, PCA was carried out based on the signal intensity of the compounds (Figure 4). The accumulative variance contribution rate of the first PC (58%) and the second PC (15%) was 73%, which could well represent the characteristic differences in the original variables. The samples were well dispersed in four different quadrants. Samples 9 to 20 and Sample 7 (Jingu 34③) were clustered on the left side of the *x*-axis, and other samples were on the right side. Samples 1 (77–322), 3 (Jingu 26), 4 (Jingu 28), 5 (Jingu 34①), and 8 (Jingu 41) were clustered together and distributed in the fourth quadrants; Samples 2 (Jingu 21), 6 (Jingu 34②), and 7 (Jingu 34③) were grouped together. Aside from Sample 16 (Jingu 59), other samples gathered together and distributed in the third quadrant. The results showed that the different varieties of foxtail millet could be clearly discriminated from one another based on the volatile compounds by HS-GC-IMS.

The differences in volatile compounds across different foxtail millet varieties were analyzed, and the volatile profile in foxtail millet is specificity-dependent on variety. As a variety widely cultivated and highly favored by consumers in northern China, Jingu21 (Sample 2) dominated in aldehydes and esters, and the typical volatile compounds were benzaldehyde (bitter almond, cherry, nut), 2-methylbutyrate (fruity, green), ethyl acetate (fruity, sweet), 2-methylbutanal (musty, nutty), and propyl acetate (raspberry-like). These typical volatile compounds combine together to give Jingu 21 a unique aroma.

### 3.3. Volatile Compounds of Foxtail Millet Analyzed by HS-SPME/GC-MS

To fully understand the variations between volatile compounds in twenty foxtail millet varieties, the volatile compounds were examined using HS-SPME/GC-MS. A total of 18 volatile compounds were tentatively identified in foxtail millet samples (Table 3), including aldehydes (6), ketones (3), alcohols (3), alkanes (4), and heterocyclic compounds (2), with hexanal, heptanal, 1-pentanol, 2-heptanone, nonanal, and 1-hexanol comprising the predominant volatile compounds determined in the 20 foxtail millet varieties under study. Compared with GC-IMS, GC-MS detected fewer volatile compounds, and most of them were macromolecular compounds.

**Figure 5:**
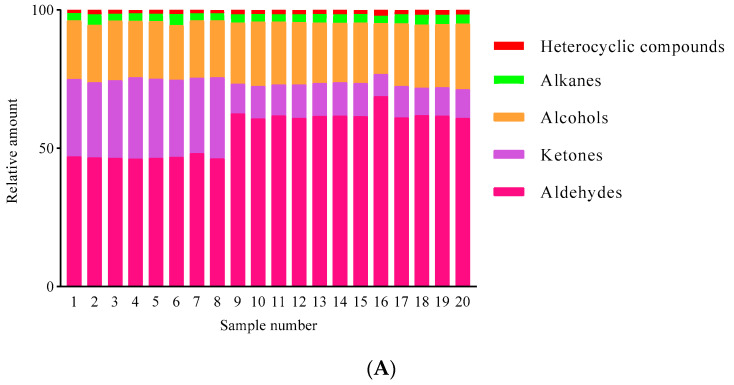

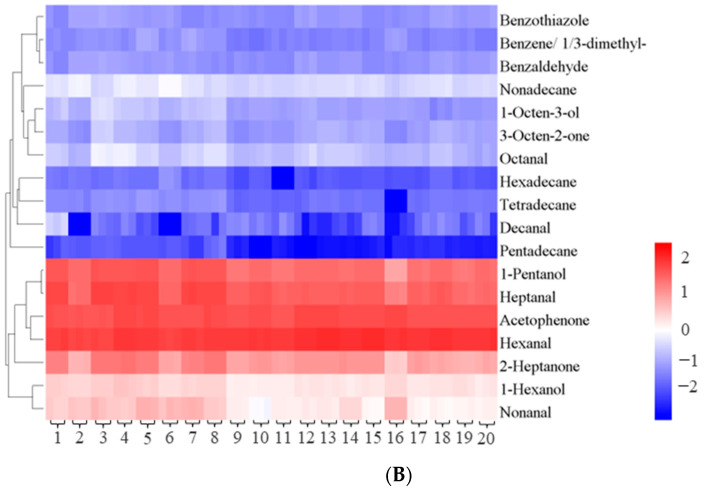
Composition (**A**) and relative content (**B**) of volatile compounds in foxtail millet.

Aldehydes generally possess green, fatty, and other odors, and play a significant role in the distinctive aroma of cereals due to their low thresholds [15,33]. Aldehydes accounted for 35.41–66.36% of the total volatile compounds (Figure 5A), and they were the main volatile components in foxtail millet. These compounds include hexanal, benzaldehyde, heptanal, octanal, nonanal and decanal, and the content of hexanal was the highest compared to other aldehydes (5.49–7.36 µg/g) (Figure 5B, Table S2). Hexanal is one of the main volatile components in grains, such as rice [16], barley [34], and oats [35]. The odor of hexanal is described as being grassy in low concentrations and an unpleasant sour smell at high concentrations [36]. The contents of heptanal and octanal were all higher than 0.22 µg/g (Figure 5B, Table S2), and they were all accompanied with a grassy and fatty odor [37,38]. Nonanal, which is generally expressed as a fragrant and fatty odor [39], accounted for 2.93–9.20% of the total volatile compounds and constitutes a common volatile substance in cereals. An earlier piece of work on the volatile profiles of foxtail millet reported that nonanal was the major volatile compound [15].

Ketones are generally considered to have a fatty and burnt aroma as well as an enhanced floral aroma with an increase in carbon chains [39]. The relative contents of ketones in foxtail millet were 8.09–29.46% (Figure 5A), of which 2-heptanone was the most abundant in Sample 8 (Jingu 41), accounting for 10.7% of the total volatile compounds (Figure 5B, Table S2). In addition, 2-heptanone can be produced by the oxidation of linoleic acid [40] with a banana and slightly medicinal odor [41], and is detected in sorghum [42], black rice [43], and other cereals.

Alcohols accounted for 18.28–23.71% of the total volatile compounds. Unsaturated alcohols have a lower threshold value and make a greater contribution to the characteristic aroma of foxtail millet. 1-octene-3-ol is an unsaturated alcohol with a mushroom and earthy aroma [17], which is considered to make an important contribution to the characteristic aroma of rice (Figure 5B, Table S2). Although a low content of 1-octene-3-ol was detected in foxtail millet (0.76–1.44%) (Figure 5A), it contributes to the characteristic aroma to foxtail millet due to its low threshold value [31].

The relative content of alkane compounds ranged from 2.66% to 3.89% (Figure 5A); however, their effects on the odor quality of the different foxtail millet were insignificant due to the high threshold. Compared with other volatile compounds, heterocyclic compounds accounted for the smallest proportion of total volatile compounds (1.12–2.13%) (Figure 5A).

Although the composition of volatile compounds in different varieties of foxtail millet was similar, the contents of volatile compounds in foxtail millet were different (Figure 5B). The contents of hexanal in Sample 12 to Sample 20 were higher than those in the other samples. Differences in the composition and content of volatile compounds contributed to the distinctive aroma of the different foxtail millet varieties.

**Figure 6:**
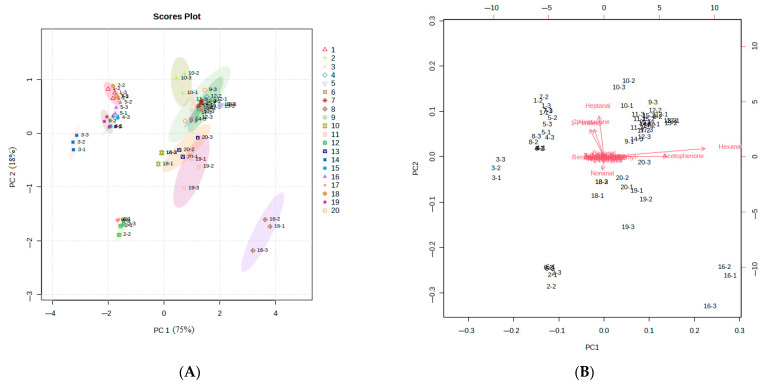
PCA analysis: score plot (**A**) and load plot (**B**).

The PCA was carried out to understand the differences in volatile compounds among 20 foxtail millet varieties (Figure 6). The cluster and separation of samples on the score plot are related to the distribution of volatile compounds on the load plot in Figure 6B. Different samples can be better resolved by presenting score plots combined with loading plots. In this way, correlations between the 18 volatile compounds and the samples can be easily observed. The score plot and loading plot revealed the volatile compounds responsible for the differences among the foxtail millet.

The accumulative variance contribution rate was 93% of the two principal components. We found that 20 varieties of foxtail millet clustered into four different groups (Figure 6A), indicating the differences in the volatile compounds of the different varieties. It was observed in the load plot that the volatile compounds with significant contributions to PC1 were hexanal and acetophenone. These compounds were characterized by green, grass, and sweet aromas. The volatile compounds with a clear contribution to PC2 were heptanal, 2-heptanone, 1-pentanol, and nonanal, which have aroma profiles of fruity, grassy, fatty, and vanilla (Table 3).

## 4. Conclusions

In this study, the characteristic volatile fingerprint of 20 foxtail millet varieties were successfully constructed by using E-nose, HS-GC-IMS, and HS-SPME/GC-MS. A total of 43 volatile compounds were identified in 20 foxtail millet samples: 34 and 18 by HS-GC-IMS and HS-SPME/GC-MS. From these, 39 volatile compounds were found in all varieties and the main volatile compounds in the foxtail millet were hexanol, hexanal, 1-pentanol, ethyl acetate, acetone, heptanal, 2-heptanone, and nonanal. Different varieties of foxtail millet usually exhibited different volatile compounds concentrations, although at similar compositions. Six volatile compounds, hexanal, heptanal, 1-pentanol, acetophenone,2-heptanone, and nonanal, contributed significantly to the volatile differences observed among the different varieties of foxtail millet. The results of this study indicate that the combination of E-nose, HS-GC-IMS, and HS-SPME/GC-MS could better retain the overall information of the volatile compounds of 20 different foxtail millets, and the application of multivariate statistical analysis made it able to distinguish foxtail millets in different varieties.

## Figures and Tables

**Table 1:** Foxtail millet samples.

No.	Varieties	No.	Varieties	No.	Varieties	No.	Varieties
1	77–322	6	Jingu 34②	11	Jingu 48	16	Jingu 59
2	Jingu 21	7	Jingu 34③	12	Jingu 53	17	Jingu 60
3	Jingu 26	8	Jingu 41	13	Jingu 54	18	Jingu 62
4	Jingu 28	9	Jingu 42	14	Jingu 55	19	Changnong35
5	Jingu 34①	10	Jingu 46	15	Jingu 58	20	Changsheng07

**Table 2:** Volatile compounds tentatively identified in the 20 foxtail millet varieties determined by HS-GC-IMS.

NO.	Compound	Aroma Description	CAS	Formula	MW	RI	Rt	Dt
1	Nonanal	cucumber	124-19-6	C_9_H_18_O	142.2	1109.2	508.010	1.48202
2	2-octenal	cucumber-like	2548-87-0	C_8_H_14_O	126.2	1054.8	429.810	1.33276
3	Octanal	orange peel, green	124-13-0	C_8_H_16_O	128.2	1003.8	356.378	1.40870
4	Hept-2-enal	sweet, marzipan	18829-55-5	C_7_H_12_O	112.2	952.9	308.696	1.25421
5	Benzaldehyde	bitter almond, cherry, nut	100-52-7	C_7_H_6_O	106.1	956.2	311.557	1.14947
6	Hex-2-enal	fruity, vegetable	505-57-7	C_6_H_10_O	98.1	849.3	233.917	1.18241
7	Hexanal	green, grassy	66-25-1	C_6_H_12_O	100.2	796.4	205.615	1.55543
8	Butanal	malty	123-72-8	C_4_H_8_O	72.1	551.7	123.358	1.27561
9	Furfural	fragrant, sweet, almond	98-01-1	C_5_H_4_O_2_	96.1	826.0	221.481	1.08335
10	2-pentenal	grassy, fruity	1576-87-0	C_5_H_8_O	84.1	747.6	184.218	1.10518
11	Pentanal	fermented bread-like	110-62-3	C_5_H_10_O	86.1	693.0	162.084	1.42118
12	2-methylbutanal	musty, nutty	96-17-3	C_5_H_10_O	86.1	662.8	153.322	1.16667
13	3-methylbutanal	sour, fatty, rancid	590-86-3	C_5_H_10_O	86.1	626.5	143.542	1.17384
14	Oct-1-en-3-ol	mushroom-like, rubbery	3191-86-4	C_8_H_16_O	128.2	980.2	332.060	1.15994
15	Hexanol	green, fruity	111-27-3	C_6_H_14_O	102.2	873.3	246.781	1.64594
16	Pentan-1-ol	oil, vanilla, sweet	71-41-0	C_5_H_12_O	88.1	769.7	193.180	1.51874
17	2-methylbutan-1-ol	fermented	137-32-6	C_5_H_12_O	88.1	741.3	181.677	1.46495
18	3-methylbutan-1-ol	oil, alcoholic, fruity	30899-19-5	C_5_H_12_O	88.1	729.3	176.779	1.48937
19	1-Propanethiol	onion	107-03-9	C_3_H_8_S	76.2	623.9	142.854	1.36308
20	Ethanol	strong alcoholic	64-17-5	C_2_H_6_O	46.1	450.5	96.080	1.13082
21	Methyl-5-hepten-2-one	banana-like, floral	110-93-0	C_8_H_14_O	126.2	988.6	339.213	1.17434
22	Dihydro-2(3h)-furanone		96-48-0	C_4_H_6_O_2_	86.1	917.7	278.655	1.08270
23	2-heptanone	fruity, spicy sweety	110-43-0	C_7_H_14_O	114.2	895.4	259.645	1.62881
24	2-Butanone	fragrant, fruit, pleasant	78-93-3	C_4_H_8_O	72.1	572.1	128.884	1.24438
25	Acetone	sweet	67-64-1	C_3_H_6_O	58.1	483.6	104.997	1.12275
26	2-Pentanone	sweety, fruity, woody	107-87-9	C_5_H_10_O	86.1	681.7	158.439	1.37005
27	Butyl acrylate	grassy, fruity	141-32-2	C_7_H_12_O_2_	128.2	899.9	263.504	1.68385
28	2-methylbutanoate	fruity, green	868-57-5	C_6_H_12_O_2_	116.2	782.4	198.326	1.18853
29	Butyl acetate	sweet, ripe banana	123-86-4	C_6_H_12_O_2_	116.2	802.6	208.924	1.61840
30	Ethyl Acetate	fruity, sweet	141-78-6	C_4_H_8_O_2_	88.1	598.4	135.960	1.33699
31	Propyl acetate	raspberry-like	109-60-4	C_5_H_10_O_2_	102.1	704.6	166.771	1.16583
32	2-pentyl furan	fruity, grassy	3777-69-3	C_9_H_14_O	138.2	990.3	340.643	1.24897
33	2-Butylfuran	mild, sweet, spicy	4466-24-4	C_8_H_12_O	124.2	890.9	256.214	1.17752
34	2-Butoxyethanol	fragrance	111-76-2	C_6_H_14_O_2_	118.2	899.4	263.075	1.20076

Note: MW: molecular mass; RI: retention index; RT: retention time; DT: drift time. The aroma description of compounds were referred from The Good Scents Company. The aroma descriptors, reported in the literature, for each volatile compound were also present.

**Table 3:** Volatile compounds tentatively identified in the 20 foxtail millet varieties determined by HS-SPME/GC-MS.

	NO.	Compound	Aroma Description	RI		CAS	Molecule Formula	Characteristicion	Identification Method
				ref	cal				
Aldehydes	1	Hexanal	green, grassy	800	796	66-25-1	C_6_H_12_O	56,72	MS,RI
	2	Benzaldehyde	bitter almond, cherry, nut	962	959	100-52-7	C_7_H_6_O	77,106	MS,RI
	3	Heptanal	rancid, pungent	901	900	111-71-7	C_7_H_14_O	70,41	MS,RI
	4	Octanal	orange peel, green	1003	1000	124-13-0	C_8_H_16_O	43,84	MS,RI
	5	Nonanal	cucumber	1104	1103	124-19-6	C_9_H_18_O	57,70	MS,RI
	6	Decanal	waxy, floral, citrus	1206	1203	112-31-2	C_10_H_20_O	43,70	MS,RI
Ketones	7	2-Heptanone	fruity, spicy sweety	891	886	110-43-0	C_7_H_14_O	43,71	MS,RI
	8	3-Octen-2-one	fruity, lemon	1040	1039	1669-44-9	C_8_H_14_O	55,111	MS,RI
	9	Acetophenone	sweet	1065	1053	98-86-2	C_8_H_8_O	105,77	MS,RI
Alcohols	10	1-Pentanol	oil, vanilla, sweet	765	755	71-41-0	C_5_H_12_O	42,70	MS,RI
	11	1-Hexanol	green, fruity	868	867	111-27-3	C_6_H_14_O	56,69	MS,RI
	12	1-Octen-3-ol	mushroom-like, rubbery	980	979	3391-86-4	C_8_H_16_O	57,72	MS,RI
Alkanes	13	Tetradecane	gasoline-like, mild waxy	1400	1398	629-59-4	C_14_H_3_0	57,71	MS,RI
	14	Pentadecane		1500	1496	629-62-9	C_15_H_32_	57,71	MS,RI
	15	Hexadecane		1600	1592	544-76-3	C_16_H_34_	57,71	MS,RI
	16	Nonadecane		1900	1885	629-92-5	C_19_H_40_	57,71	MS,RI
Heterocyclic compounds	17	Benzothiazole	rose, vegetable	1229	1224	95-16-9	C_7_H_5_NS	135,108	MS,RI
	18	Benzene, 1,3-dimethyl-		866	855	108-38-3	C_8_H_10_	91,106	MS,RI

Note: ref: RI literature value; cal: RI calculated value. Identification method: MS, mass spectrum comparison using NIST14 library; RI: RI calculation in agreement with literature value. The aroma description of compounds were referred from The Good Scents Company Information System.

## Data Availability

Data are contained within the article or Supplementary Material.

## Supplementary Materials

The following supporting information can be downloaded at: https://www.mdpi.com/article/10.3390/foods12234273/s1, Figure S1: Chromatograms of the 20 foxtail millet varieties by E-Nose analysis, Table S1: The signal intensities of volatile compounds in 20 varieties of foxtail millet analyzed by HS-GC-IMS, Table S2: Contents (mean ± standard deviation) of the volatile compounds tentatively identified in 20 varieties of foxtail millet by HS-SPME/GC-MS.
