# Supplementary material for: Volatile Fingerprint and Differences in Volatile Compounds of Different Foxtail Millet (Setaria italica Beauv.) Varieties
Source: Foods. 2023 Nov 27;12(23):4273. doi: 10.3390/foods12234273 (PMC10705982; doi:10.3390/foods12234273)
Supplement: Supplementary file 1 [file foods-12-04273-s001.zip › foods-2677382-supplementary.pdf]

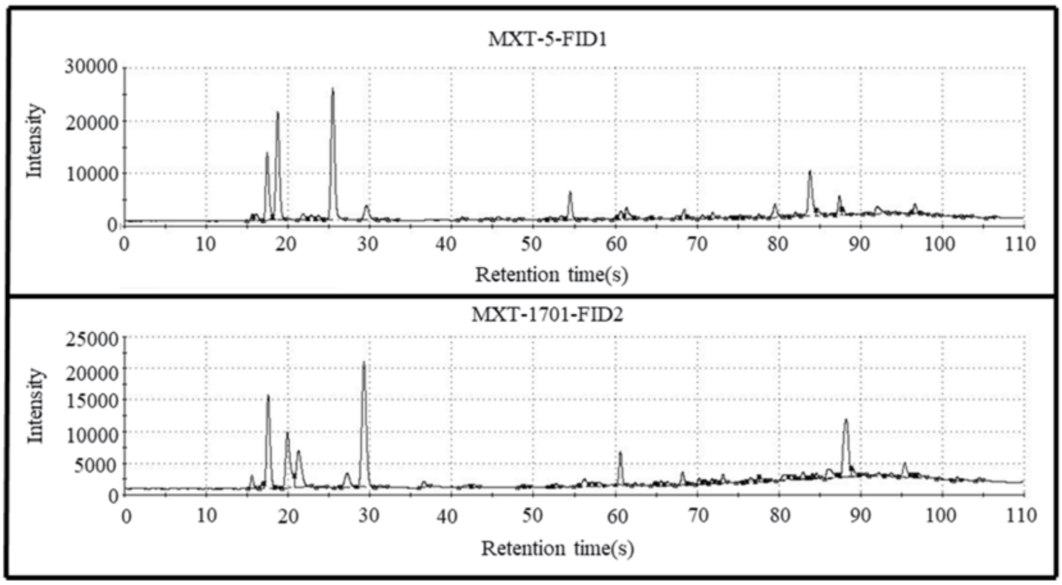
MXT-5-FID1
Intensity
30000
20000
10000
0
0 10 20 30 40 50 60 70 80 90 100 110
Retention time(s)
MXT-1701-FID2
Intensity
25000
20000
15000
10000
5000
0
0 10 20 30 40 50 60 70 80 90 100 110
Retention time(s)

1.

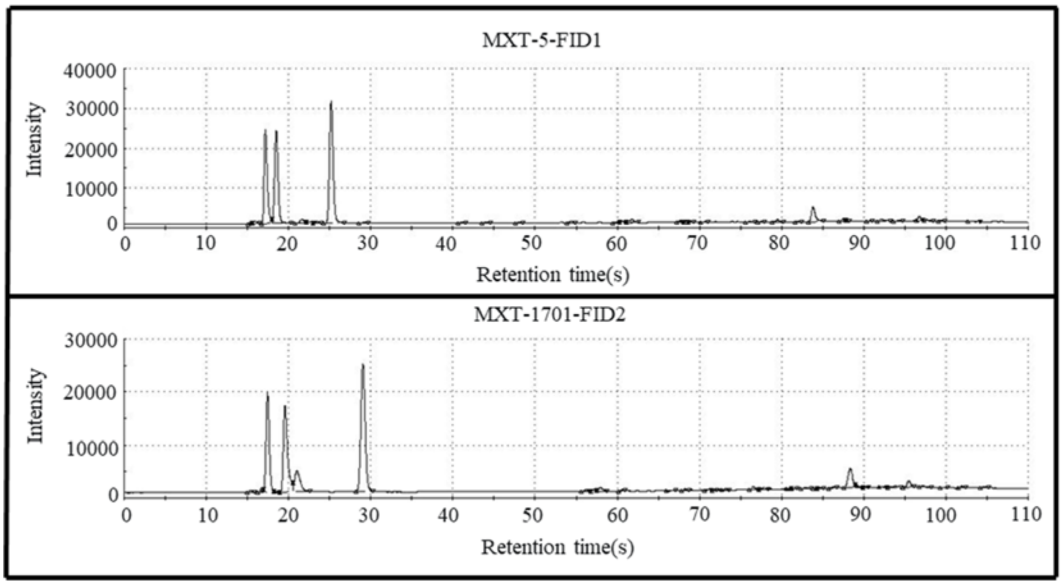
MXT-5-FID1
Intensity
40000
30000
20000
10000
0
0 10 20 30 40 50 60 70 80 90 100 110
Retention time(s)
MXT-1701-FID2
Intensity
30000
20000
10000
0
0 10 20 30 40 50 60 70 80 90 100 110
Retention time(s)

2.

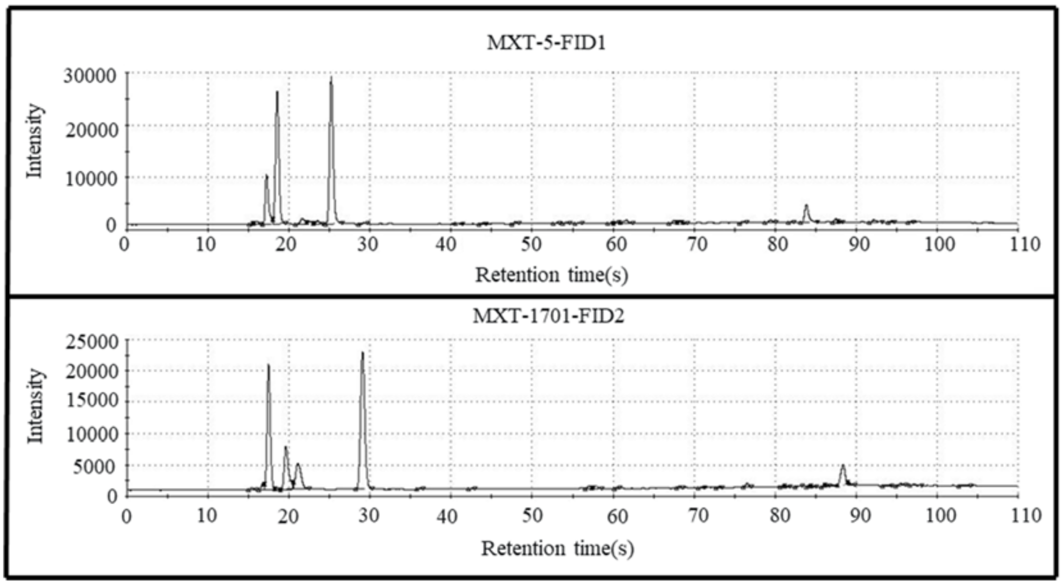
MXT-5-FID1
Intensity
30000
20000
10000
0
0 10 20 30 40 50 60 70 80 90 100 110
Retention time(s)
MXT-1701-FID2
Intensity
25000
20000
15000
10000
5000
0
0 10 20 30 40 50 60 70 80 90 100 110
Retention time(s)

3.

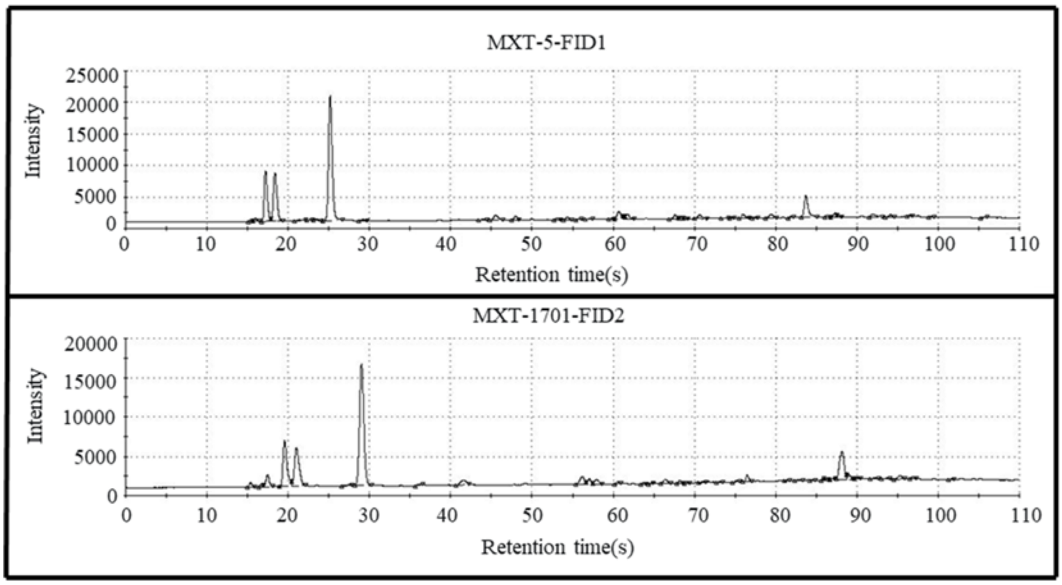
MXT-5-FID1
Intensity
Retention time(s)
MXT-1701-FID2
Intensity
Retention time(s)

4.

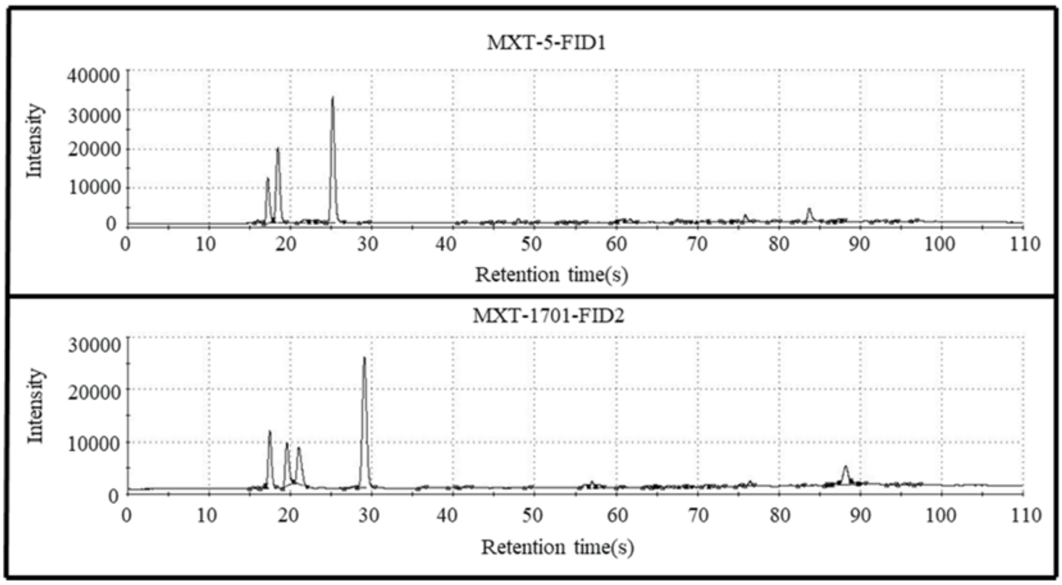
MXT-5-FID1
Intensity
Retention time(s)
MXT-1701-FID2
Intensity
Retention time(s)

5.

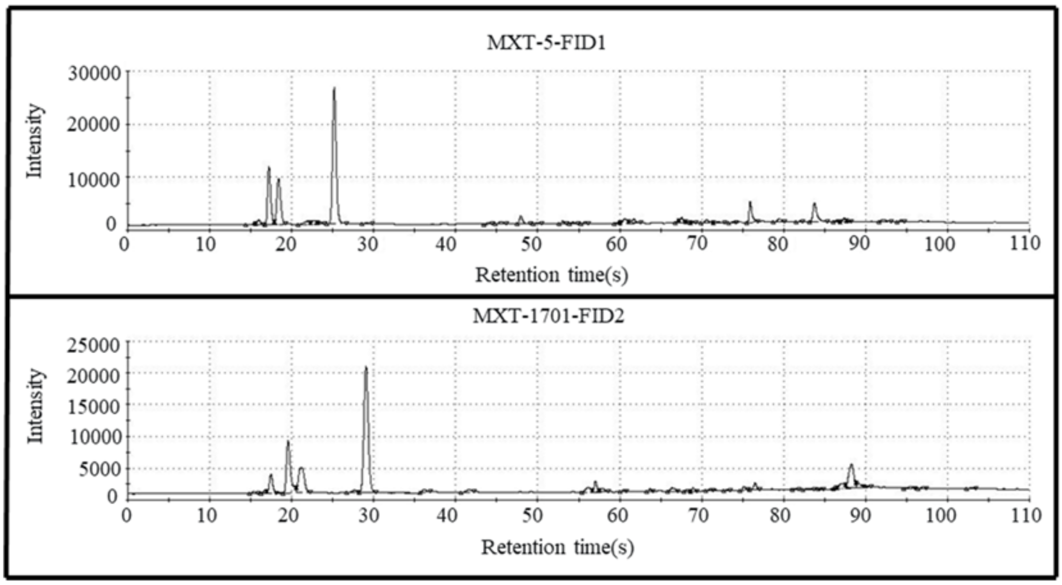
MXT-5-FID1
Intensity
Retention time(s)
MXT-1701-FID2
Intensity
Retention time(s)

6.

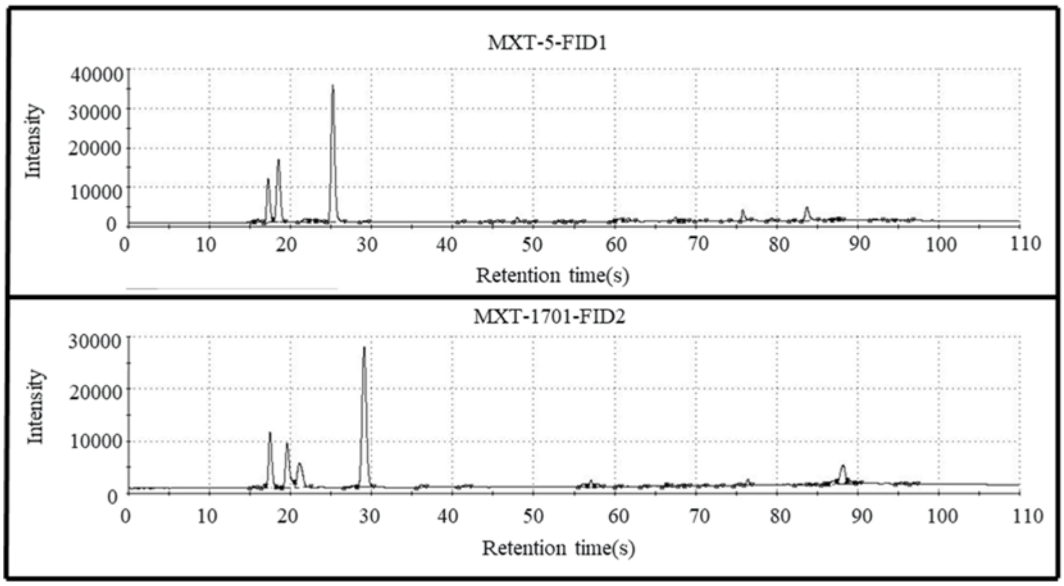
MXT-5-FID1
Intensity
40000
30000
20000
10000
0
0 10 20 30 40 50 60 70 80 90 100 110
Retention time(s)
MXT-1701-FID2
Intensity
30000
20000
10000
0
0 10 20 30 40 50 60 70 80 90 100 110
Retention time(s)

7.

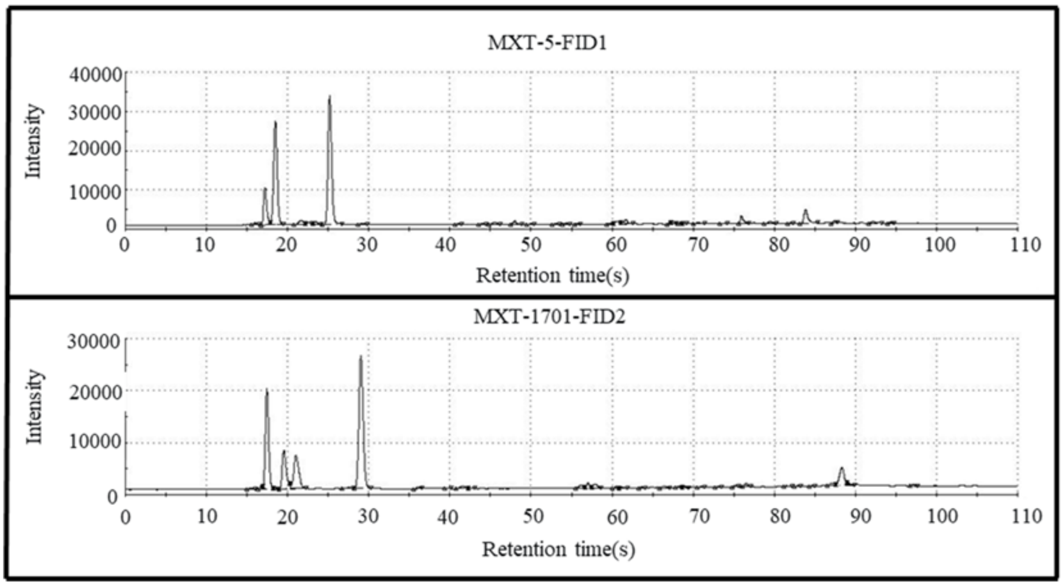
MXT-5-FID1
Intensity
40000
30000
20000
10000
0
0 10 20 30 40 50 60 70 80 90 100 110
Retention time(s)
MXT-1701-FID2
Intensity
30000
20000
10000
0
0 10 20 30 40 50 60 70 80 90 100 110
Retention time(s)

8.

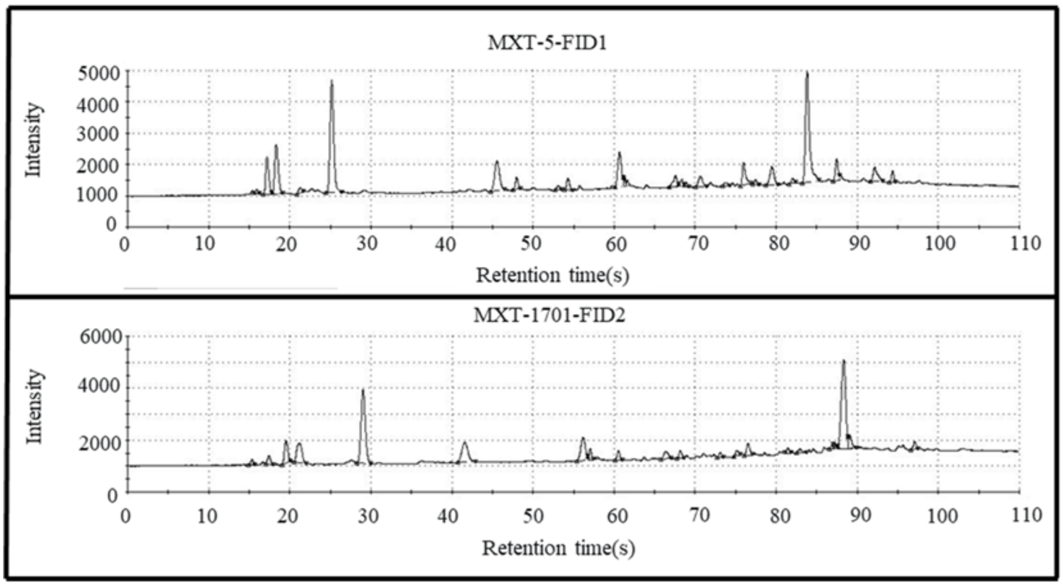
MXT-5-FID1
Intensity
5000
4000
3000
2000
1000
0
0 10 20 30 40 50 60 70 80 90 100 110
Retention time(s)
MXT-1701-FID2
Intensity
6000
4000
2000
0
0 10 20 30 40 50 60 70 80 90 100 110
Retention time(s)

9.

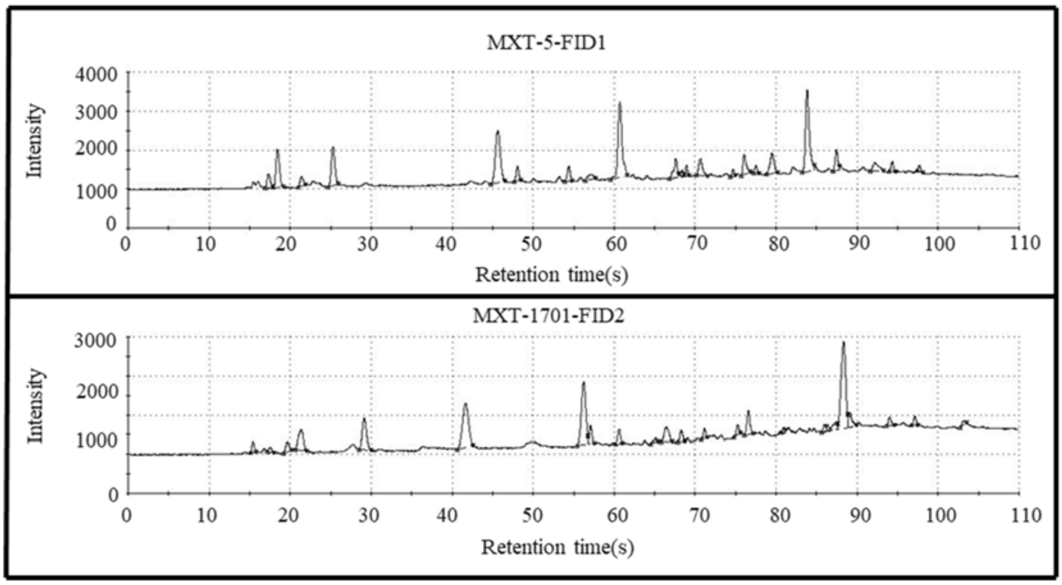
MXT-5-FID1
Intensity
4000
3000
2000
1000
0
0 10 20 30 40 50 60 70 80 90 100 110
Retention time(s)
MXT-1701-FID2
Intensity
3000
2000
1000
0
0 10 20 30 40 50 60 70 80 90 100 110
Retention time(s)

10.

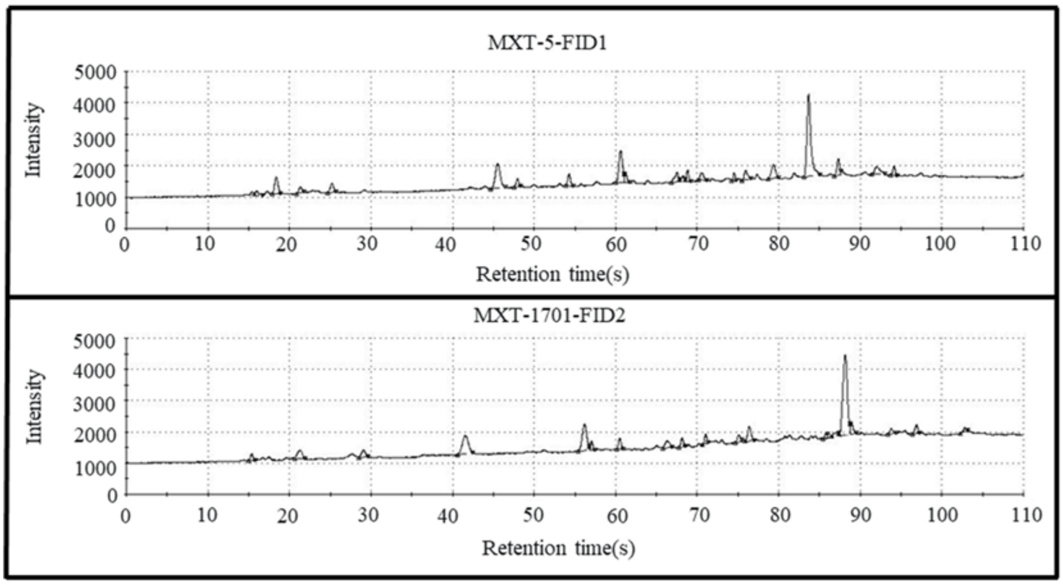
MXT-5-FID1
Intensity
5000
4000
3000
2000
1000
0
0 10 20 30 40 50 60 70 80 90 100 110
Retention time(s)
MXT-1701-FID2
Intensity
5000
4000
3000
2000
1000
0
0 10 20 30 40 50 60 70 80 90 100 110
Retention time(s)

11.

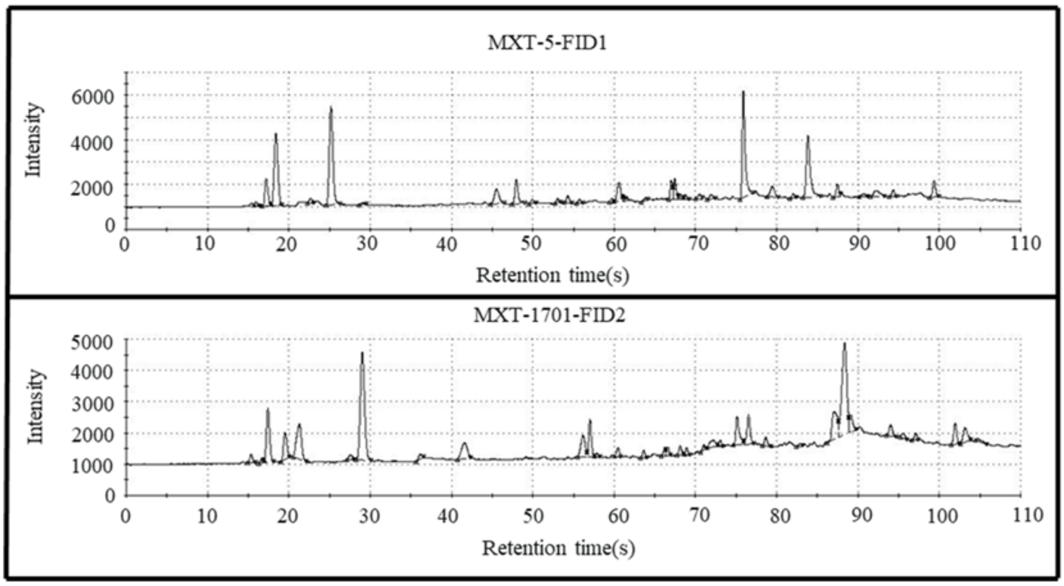
MXT-5-FID1
Intensity
6000
4000
2000
0
0 10 20 30 40 50 60 70 80 90 100 110
Retention time(s)
MXT-1701-FID2
Intensity
5000
4000
3000
2000
1000
0
0 10 20 30 40 50 60 70 80 90 100 110
Retention time(s)

12.

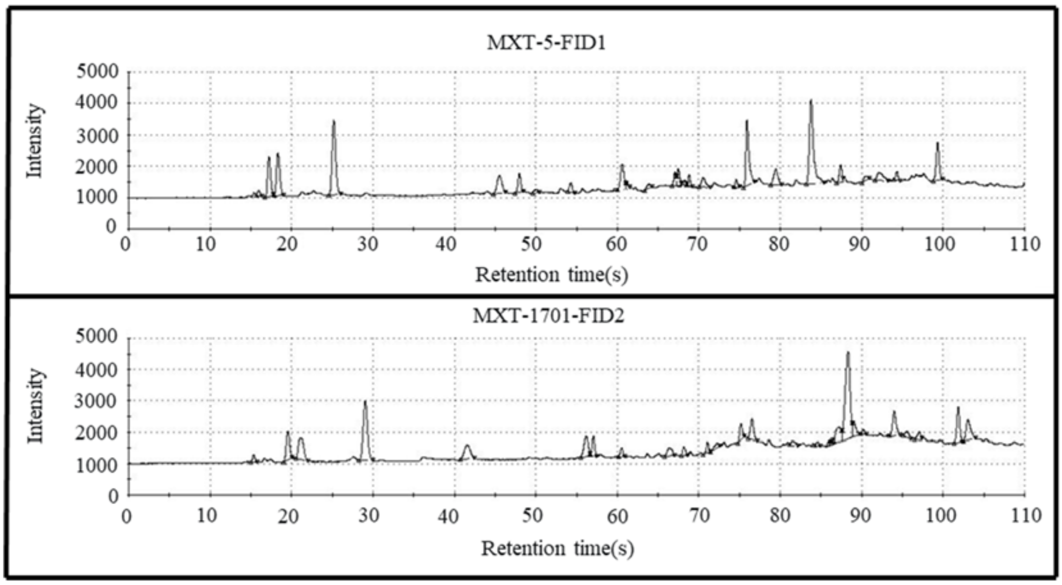
MXT-5-FID1
Intensity
5000
4000
3000
2000
1000
0
0 10 20 30 40 50 60 70 80 90 100 110
Retention time(s)
MXT-1701-FID2
Intensity
5000
4000
3000
2000
1000
0
0 10 20 30 40 50 60 70 80 90 100 110
Retention time(s)
13.

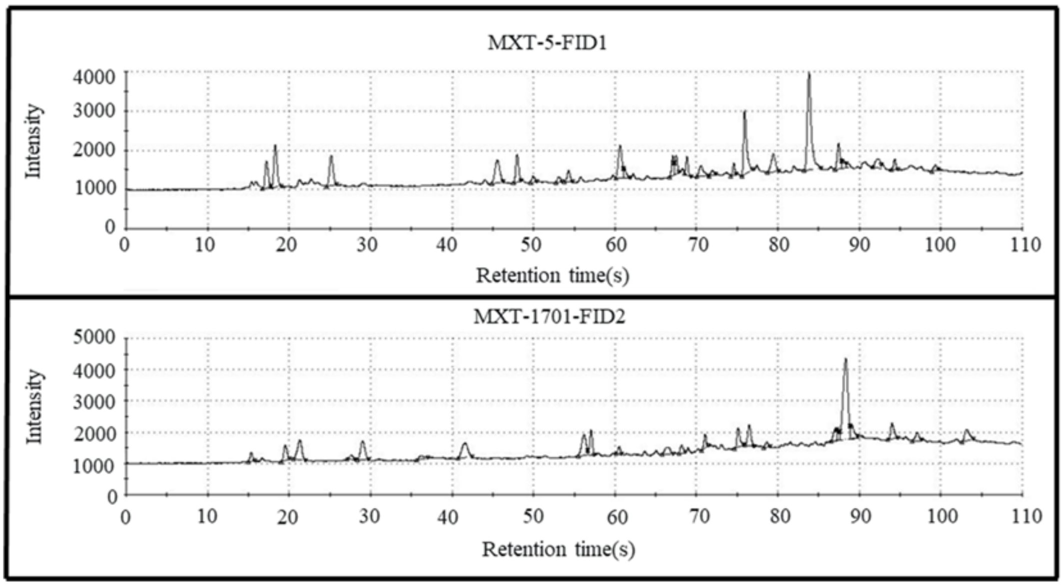
MXT-5-FID1
Intensity
4000
3000
2000
1000
0
0 10 20 30 40 50 60 70 80 90 100 110
Retention time(s)
MXT-1701-FID2
Intensity
5000
4000
3000
2000
1000
0
0 10 20 30 40 50 60 70 80 90 100 110
Retention time(s)
14.

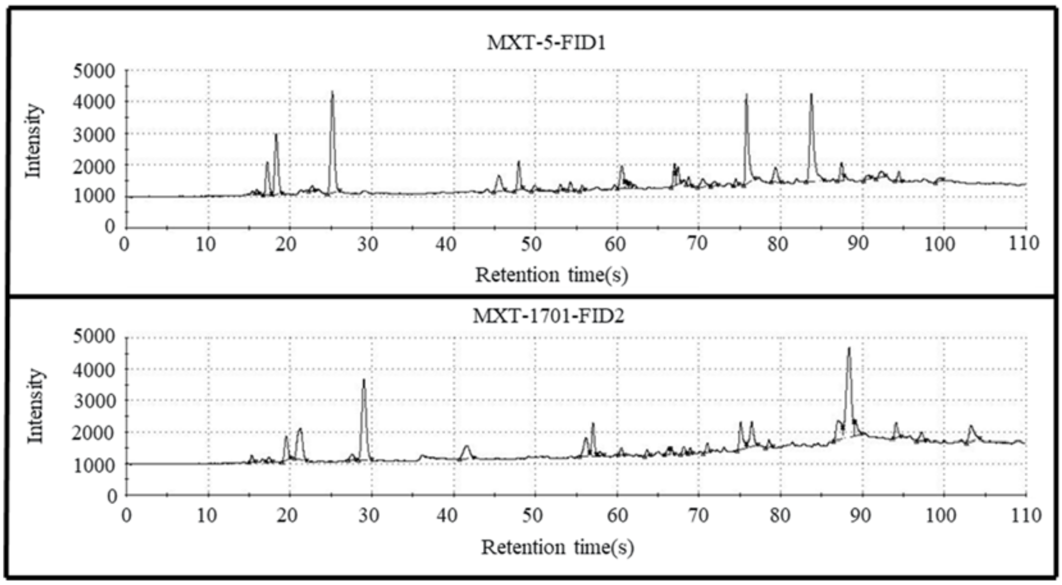
MXT-5-FID1
Intensity
5000
4000
3000
2000
1000
0
0 10 20 30 40 50 60 70 80 90 100 110
Retention time(s)
MXT-1701-FID2
Intensity
5000
4000
3000
2000
1000
0
0 10 20 30 40 50 60 70 80 90 100 110
Retention time(s)
15.

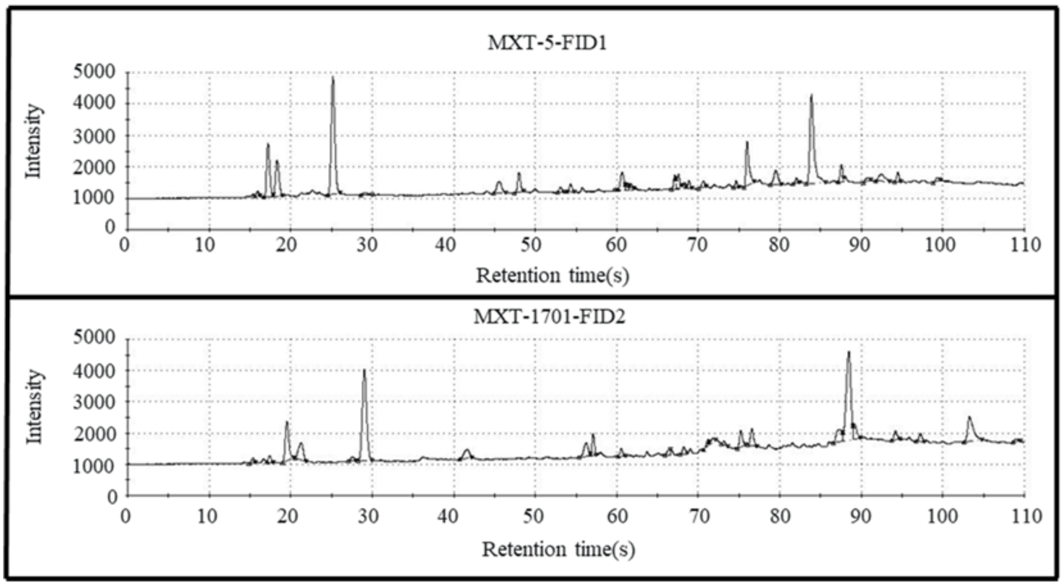
MXT-5-FID1
Intensity
Retention time(s)
MXT-1701-FID2
Intensity
Retention time(s)

16.

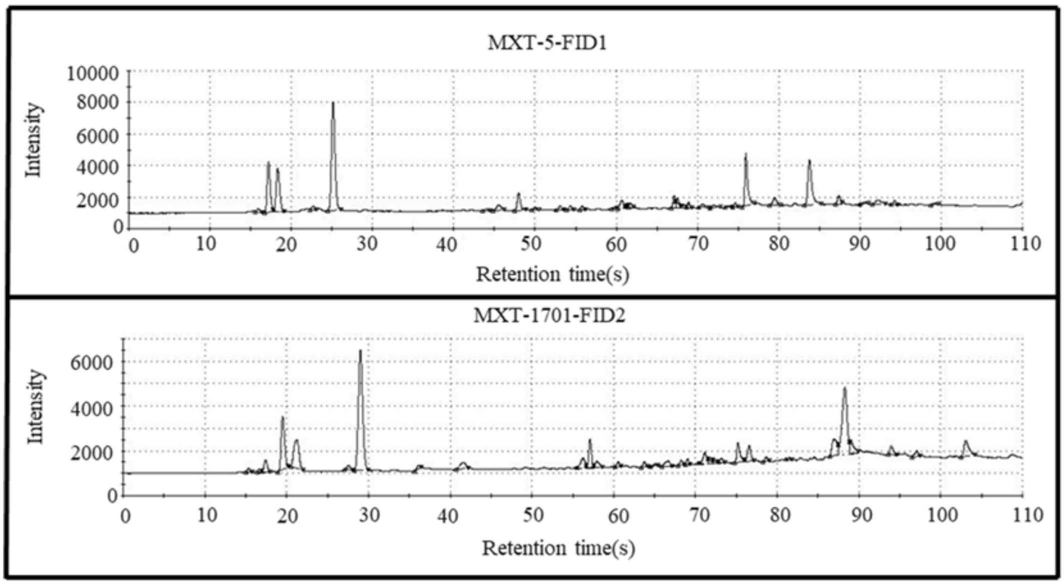
MXT-5-FID1
Intensity
Retention time(s)
MXT-1701-FID2
Intensity
Retention time(s)

17.

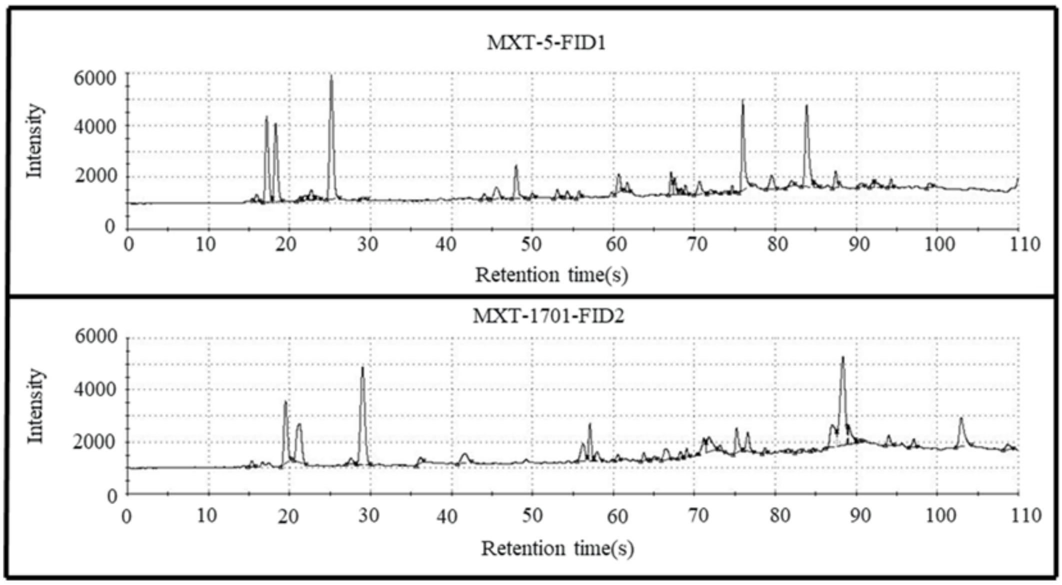
MXT-5-FID1
Intensity
Retention time(s)
MXT-1701-FID2
Intensity
Retention time(s)

18.

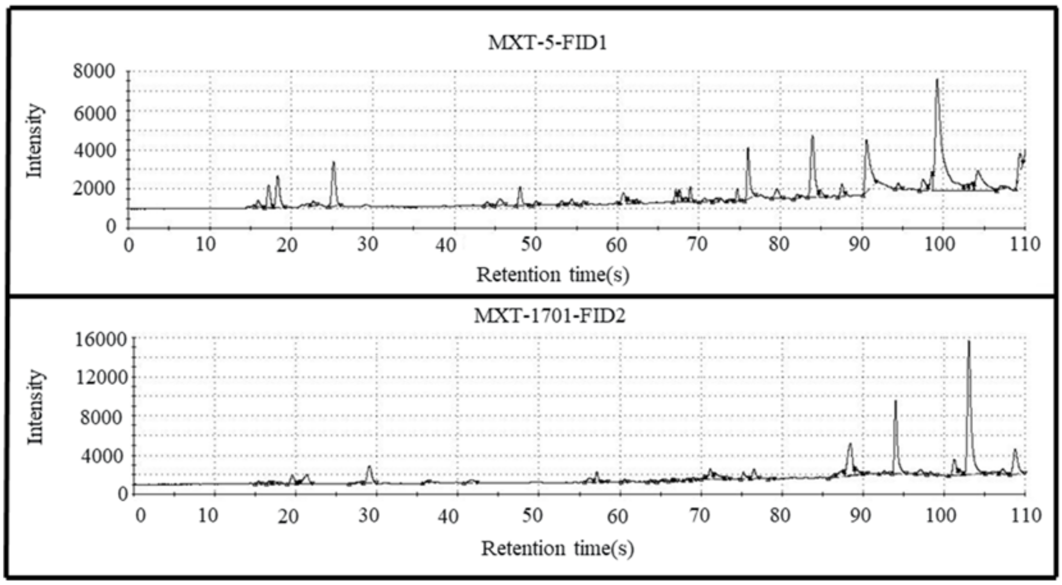


19.

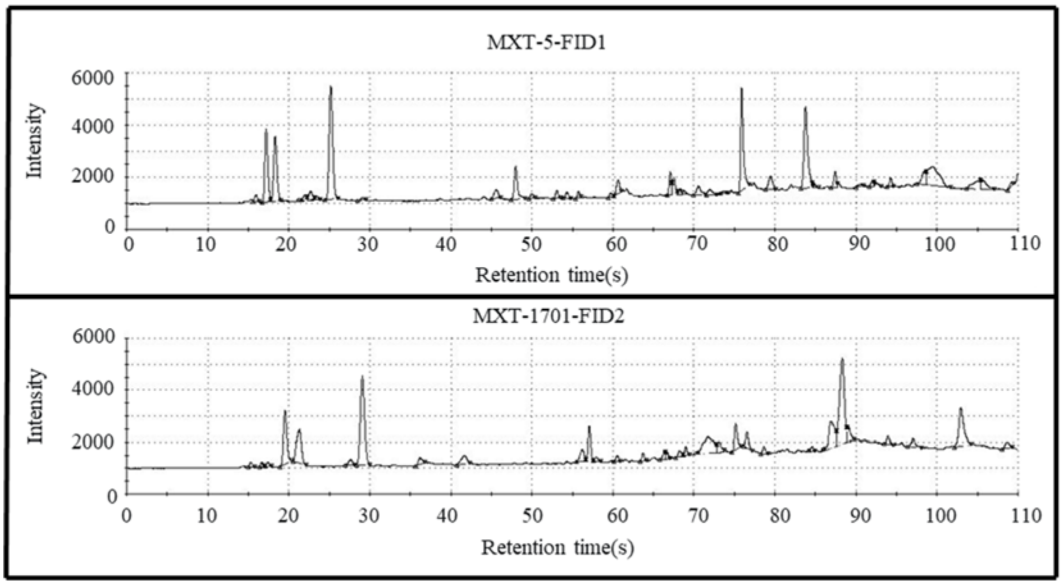


20.

**Figure S1**. Chromatograms of the 20 foxtail millet varieties by E-Nose analysis.

**Table S1.** The signal intensities of volatile compounds in 20 varieties of foxtail millet analyzed by HS-GC-IMS.

| No. | Compound | Peak intensity | | | | | | | | | |
|---|---|---|---|---|---|---|---|---|---|---|---|
| | | 77-322 | Jingu 21 | Jingu 26 | Jingu 28 | Jingu 34① | Jingu 34② | Jingu 34③ | Jingu 41 | Jingu 42 | Jingu 46 |
| 1 | Nonanal | 710.45 ±44.96[a] | 806.43 ±28.09 | 860.28 ±90.20 | 746.90 ±22.44 | 999.55 ±39.88 | 908.92 ±71.62 | 847.96 ±186.17 | 752.35 ±36.73 | 599.85 ±175.25 | 372.84±9.98 |
| 2 | 2-octenal | 726.08 ±8.03 | 491.14 ±27.94 | 771.95 ±10.97 | 756.37 ±32.76 | 701.37 ±10.89 | 502.01 ±11.26 | 471.87 ±7.98 | 681.62 ±20.88 | 643.37 ±44.33 | 730.30±1.50 |
| 3 | 1 | 171.70 ±6.34 | 154.67 ±5.28 | 169.53 ±8.47 | 177.35 ±2.63 | 230.41 ±7.85 | 136.91 ±3.20 | 96.93 ±3.29 | 149.25 ±10.70 | 110.29 ±10.75 | 123.96±7.07 |
| 4 | 2 | 378.97 ±5.20 | 255.70 ±15.82 | 527.44 ±4.23 | 443.79 ±1.20 | 363.21 ±7.46 | 277.39 ±10.23 | 324.54 ±19.71 | 420.14 ±7.84 | 300.66 ±7.74 | 286.82±9.08 |
| 5 | Octanal | 212.55 ±10.63 | 161.27 ±3.41 | 335.68 ±13.21 | 326.24 ±13.93 | 238.29 ±4.12 | 182.27 ±3.97 | 170.04 ±9.32 | 275.94 ±3.42 | 170.16 ±16.87 | 180.86±8.65 |
| 6 | 2-pentyl furan | 1126.34 ±14.77 | 693.97 ±15.97 | 1410.12 ±15.17 | 1170.76 ±19.63 | 1234.00 ±5.28 | 734.47 ±5.95 | 583.54 ±43.51 | 1229.27 ±11.41 | 1033.40 ±98.03 | 1292.22±17.61 |
| 7 | hept-2-enal-M | 709.44 ±12.61 | 641.57 ±0.85 | 723.95 ±10.69 | 798.27 ±11.06 | 704.35 ±4.46 | 596.84 ±4.92 | 697.26 ±20.35 | 687.44 ±11.98 | 536.03 ±70.78 | 499.74±16.89 |
| 8 | hept-2-enal-D | 224.73 ±16.13 | 140.55 ±6.99 | 317.16 ±10.66 | 366.05 ±6.19 | 272.48 ±1.98 | 137.97 ±3.77 | 217.05 ±16.61 | 270.53 ±5.70 | 130.17 ±50.98 | 141.28±3.57 |
| 9 | methyl-5-hepten-2-one | 174.46±1.15 | 116.32±4.93 | 133.99±5.49 | 132.75±2.31 | 161.31±15.10 | 151.83±4.49 | 113.52±14.22 | 139.98±8.17 | 104.39±40.40 | 100.62±4.95 |
| 10 | oct-1-en-3-ol | 188.17±28.69 | 146.11±9.16 | 265.00±5.34 | 211.30±4.04 | 192.83±8.33 | 156.61±5.62 | 134.49±3.50 | 216.60±10.49 | 144.43±37.02 | 135.43±1.76 |
| 11 | Benzaldehyde | 100.55±10.55 | 136.52±2.67 | 139.19±0.31 | 128.54±7.03 | 117.26±3.88 | 120.36±4.63 | 79.38±2.58 | 105.79±4.16 | 103.77±8.63 | 102.00±4.11 |

| | | | | | | | | | | | |
|---|---|---|---|---|---|---|---|---|---|---|---|
| 12 | dihydro-2(3h)-furanone | 133.42±11.49 | 140.15±10.08 | 202.26±31.87 | 174.65±30.74 | 107.92±1.48 | 80.08±14.75 | 127.45±15.50 | 132.24±20.42 | 98.43±59.36 | 67.55±3.70 |
| 13 | 3 | 160.63±7.84 | 109.15±16.43 | 222.58±28.73 | 179.19±25.27 | 119.52±20.14 | 72.16±11.71 | 85.23±8.28 | 141.74±22.19 | 103.78±46.61 | 92.01±1.62 |
| 14 | Butyl acrylate | 452.36±9.78 | 341.00±16.20 | 560.35±16.78 | 482.15±8.06 | 363.08±10.22 | 327.99±3.89 | 327.18±24.25 | 472.74±1.30 | 358.43±10.44 | 322.19±7.81 |
| 15 | 2-heptanone | 1804.39±16.38 | 861.16±31.76 | 1977.91±22.53 | 2070.14±31.42 | 1918.66±12.40 | 1148.75±15.04 | 850.04±57.60 | 1995.55±21.46 | 1262.35±190.00 | 1349.99±32.93 |
| 16 | n-Hexanol | 3483.37±63.69 | 1895.46±34.43 | 3602.76±40.69 | 3544.14±28.40 | 3428.88±27.32 | 2362.77±9.66 | 2302.53±127.02 | 3459.65±56.73 | 2791.72±409.63 | 2929.69±105.52 |
| 17 | hex-2-enal-M | 296.38±11.31 | 318.39±5.23 | 335.43±9.36 | 316.78±5.64 | 297.85±4.43 | 302.25±5.00 | 268.09±11.96 | 294.17±10.04 | 218.46±31.16 | 176.68±3.77 |
| 18 | hex-2-enal-D | 244.18±15.24 | 172.41±3.03 | 342.14±4.64 | 315.17±5.00 | 301.39±1.62 | 193.51±3.43 | 132.37±12.74 | 288.65±5.93 | 172.12±13.15 | 186.69±13.25 |
| 19 | 2-Butoxyethanol | 83.72±2.83 | 66.35±4.14 | 78.67±3.30 | 72.23±2.41 | 100.66±7.09 | 79.93±3.89 | 43.08±3.11 | 105.67±2.83 | 52.70±17.00 | 53.04±5.32 |
| 20 | 2-n-Butylfuran | 92.23±1.43 | 65.42±1.76 | 126.78±1.78 | 92.70±2.00 | 102.70±3.09 | 65.44±0.43 | 48.48±2.55 | 96.10±3.25 | 97.66±9.42 | 120.13±4.52 |
| 21 | 4 | 99.03±1.81 | 92.80±6.67 | 108.15±2.81 | 104.08±2.20 | 115.69±3.99 | 103.32±2.07 | 62.70±1.93 | 112.54±5.88 | 80.41±27.77 | 70.56±1.08 |
| 22 | Furfural | 41.12±9.56 | 26.98±1.92 | 29.49±3.46 | 26.63±0.16 | 24.67±1.71 | 21.22±1.89 | 27.71±1.00 | 22.80±2.21 | 29.23±4.18 | 20.80±0.56 |
| 23 | 5 | 41.22±4.11 | 45.14±1.09 | 58.73±2.12 | 50.88±1.85 | 43.04±1.11 | 45.23±1.16 | 33.94±2.99 | 65.45±5.82 | 39.18±9.23 | 31.46±0.98 |
| 24 | methyl 2-methylbutanoate | 63.29±3.05 | 82.30±1.84 | 58.07±2.26 | 70.42±0.27 | 67.26±2.10 | 87.29±2.39 | 72.02±2.16 | 71.62±4.30 | 43.48±15.51 | 32.99±3.59 |

| | | | | | | | | | | | |
|---|---|---|---|---|---|---|---|---|---|---|---|
| 25 | Hexanal | 3118.64±113.89 | 2925.05±97.98 | 3514.26±101.18 | 3481.10±47.07 | 3339.54±33.45 | 3013.49±12.76 | 2666.41±14.39 | 3262.28±97.59 | 2982.34±261.07 | 3263.24±24.21 |
| 26 | pentan-1-ol | 2944.64±34.51 | 1632.92±13.60 | 2970.30±65.14 | 2944.85±17.59 | 3038.16±23.96 | 2264.27±7.45 | 1661.84±55.55 | 2938.23±46.68 | 2214.95±353.03 | 2313.50±20.52 |
| 27 | 6 | 48.10±10.91 | 56.97±1.72 | 63.64±3.00 | 57.02±4.49 | 54.42±0.68 | 56.58±3.77 | 44.35±6.27 | 70.87±10.77 | 46.77±28.75 | 25.87±1.71 |
| 28 | Butyl acetate | 86.79±5.15 | 50.49±1.97 | 53.29±2.44 | 59.16±2.01 | 72.33±4.87 | 47.93±1.77 | 63.82±5.35 | 59.03±1.63 | 61.62±23.91 | 47.77±2.60 |
| 29 | 7 | 103.40±7.07 | 168.80±8.34 | 111.54±3.35 | 102.90±5.75 | 142.24±4.63 | 105.99±4.14 | 131.45±13.83 | 114.83±2.38 | 88.03±13.15 | 78.14±5.73 |
| 30 | 8 | 83.19±13.99 | 73.40±4.53 | 138.75±1.00 | 64.01±2.05 | 85.49±3.52 | 70.79±3.57 | 57.97±1.64 | 97.48±8.98 | 98.14±12.79 | 74.81±4.84 |
| 31 | 2-pentenal-M | 51.44±7.50 | 52.58±2.32 | 56.04±1.11 | 56.02±1.83 | 55.69±0.92 | 52.07±2.03 | 38.89±3.13 | 56.49±0.68 | 39.16±13.30 | 28.24±1.53 |
| 32 | 9 | 77.45±4.81 | 80.53±1.55 | 72.74±0.95 | 80.42±4.82 | 73.76±1.53 | 107.88±5.54 | 44.32±2.50 | 78.78±0.93 | 52.41±5.06 | 59.96±1.53 |
| 33 | 2-methylbutan-1-ol | 319.45±7.90 | 275.87±14.56 | 468.77±6.90 | 387.74±3.78 | 382.48±6.34 | 332.69±8.77 | 261.78±17.22 | 377.84±6.33 | 216.60±24.12 | 291.66±10.38 |
| 34 | 3-methylbutan-1-ol | 520.78±21.10 | 525.77±28.00 | 528.14±4.69 | 508.44±5.15 | 754.66±2.83 | 469.46±5.37 | 647.57±20.78 | 381.57±19.81 | 402.58±26.10 | 424.88±15.86 |
| 35 | n-Propyl acetate-D | 128.85±4.20 | 119.77±5.89 | 56.31±1.56 | 112.76±1.04 | 208.53±0.63 | 141.87±7.49 | 81.46±8.75 | 169.42±6.80 | 106.78±44.15 | 50.79±2.58 |
| 36 | Pentanal | 268.18±13.40 | 253.07±1.96 | 419.61±6.02 | 424.62±10.54 | 459.19±2.51 | 319.86±7.83 | 114.19±4.41 | 361.05±9.16 | 188.87±8.27 | 259.07±11.29 |
| 37 | 1-Propanethiol | 340.84±9.93 | 302.33±12.05 | 416.70±9.31 | 266.37±3.06 | 320.36±6.99 | 256.94±2.92 | 428.95±13.50 | 301.74±31.90 | 293.99±7.59 | 297.97±9.79 |
| 38 | Ethyl Acetate | 3542.74±39.77 | 3994.56±101.51 | 2606.74±17.94 | 2824.99±54.15 | 3147.94±5.39 | 3454.90±18.02 | 2420.35±83.52 | 3606.46±46.98 | 2627.90±590.28 | 1751.05±27.70 |
| 39 | 2-Butanone | 1062.75±36.06 | 829.47±16.71 | 976.93±13.43 | 1256.15±25.40 | 1059.58±7.65 | 890.63±2.65 | 808.15±36.22 | 987.65±18.82 | 679.74±61.03 | 711.10±3.12 |

| | | | | | | | | | | | |
|---|---|---|---|---|---|---|---|---|---|---|---|
| 40 | acetone | 3753.39±132.78 | 3606.79±64.51 | 2974.67±29.64 | 4124.14±58.51 | 3946.33±50.90 | 3564.16±71.62 | 3362.14±57.44 | 3954.88±28.73 | 3789.47±307.37 | 4037.20±10.47 |
| 41 | ethanol | 1314.63±24.25 | 1141.42±18.82 | 1194.20±6.97 | 1350.60±63.40 | 1235.04±16.10 | 1165.63±33.90 | 1327.65±50.54 | 1343.62±26.64 | 1176.25±107.57 | 1112.72±36.28 |
| 42 | 10 | 1368.38±9.45 | 917.65±13.56 | 1477.69±57.34 | 1192.03±23.79 | 1090.04±23.88 | 993.76±32.86 | 1414.79±37.39 | 1244.82±58.16 | 1440.07±62.10 | 1377.13±66.91 |
| 43 | 2-methylbutanal | 163.80±4.96 | 211.44±6.72 | 131.25±7.12 | 128.63±2.13 | 141.52±3.03 | 181.92±4.91 | 193.95±8.50 | 135.05±4.52 | 158.38±24.71 | 169.61±2.73 |
| 44 | 3-methylbutanal | 280.48±14.19 | 320.95±12.62 | 248.55±11.08 | 201.43±7.78 | 253.83±9.55 | 285.63±15.12 | 256.04±8.89 | 240.80±38.83 | 276.94±52.69 | 303.66±10.11 |
| 45 | 2-Pentanone | 201.72±4.58 | 115.63±1.69 | 181.56±8.03 | 259.89±4.66 | 252.46±2.76 | 165.85±4.97 | 107.05±8.01 | 179.21±7.92 | 94.50±16.47 | 95.12±6.07 |
| 46 | butanal | 152.44±13.44 | 166.68±2.53 | 232.04±3.24 | 163.06±5.78 | 158.60±5.29 | 144.77±2.17 | 152.69±0.28 | 190.88±23.94 | 146.15±3.67 | 129.04±6.58 |
| 47 | 11 | 144.02±2.07 | 104.92±5.86 | 227.27±7.75 | 173.52±2.84 | 148.79±2.14 | 109.81±3.50 | 85.85±5.99 | 178.92±3.55 | 109.44±15.41 | 117.21±5.83 |
| 48 | 2-pentenal-D | 83.54±5.79 | 72.97±3.91 | 123.66±2.89 | 86.63±1.61 | 85.72±0.73 | 57.22±3.34 | 55.88±3.08 | 94.00±5.29 | 74.12±7.20 | 55.14±2.49 |
| 49 | 12 | 285.07±11.33 | 344.33±1.87 | 244.45±6.79 | 324.93±10.47 | 297.41±1.66 | 369.81±3.38 | 285.89±2.24 | 251.60±15.31 | 221.80±8.92 | 246.33±8.44 |
| 50 | n-Propyl acetate-M | 142.61±1.95 | 148.02±1.09 | 85.04±2.75 | 95.41±1.75 | 148.07±2.78 | 143.20±6.01 | 156.38±4.44 | 126.39±6.19 | 112.99±7.75 | 93.44±1.06 |

| No. | Compound | Peak intensity | | | | | | | | | |
|---|---|---|---|---|---|---|---|---|---|---|---|
| | | **Jingu 48** | **Jingu 53** | **Jingu 54** | **Jingu 55** | **Jingu 58** | **Jingu 59** | **Jingu 60** | **Jingu 62** | **Changnong35** | **Changsheng 07** |
| 1 | Nonanal | 492.21±8.16 | 552.59±94.27 | 522.58±21.30 | 639.57±15.51 | 431.17±25.80 | 975.41±19.77 | 457.88±37.55 | 456.32±28.06 | 463.55±12.07 | 466.02±20.24 |
| 2 | 2-octenal | 665.61±8.68 | 750.10±68.03 | 697.40±16.94 | 694.65±24.49 | 694.64±5.12 | 588.62±4.23 | 589.14±4.96 | 646.44±11.54 | 554.85±9.62 | 529.46±7.16 |
| 3 | 1 | 106.14±5.02 | 141.84±17.18 | 129.11±5.19 | 133.25±2.94 | 122.85±3.63 | 112.92±2.45 | 121.50±2.44 | 168.71±6.13 | 113.06±6.43 | 118.87±6.14 |
| 4 | 2 | 294.82±7.78 | 355.97±32.76 | 347.62±8.99 | 317.99±9.49 | 331.45±6.16 | 378.88±13.73 | 298.45±1.54 | 294.12±3.95 | 311.59±8.15 | 288.77±15.03 |
| 5 | Octanal | 172.60±3.31 | 229.47±27.38 | 213.25±1.65 | 214.05±4.26 | 178.51±6.70 | 157.57±7.31 | 164.65±9.02 | 198.33±3.77 | 152.56±11.37 | 139.64±11.93 |
| 6 | 2-pentyl furan | 1054.16±13.14 | 1424.16±253.03 | 1286.25±13.69 | 1281.10±21.63 | 1145.52±14.54 | 866.75±32.96 | 1111.61±40.68 | 1438.70±12.33 | 946.49±34.11 | 932.94±16.54 |
| 7 | hept-2-enal-M | 501.41±4.20 | 559.40±17.17 | 559.00±12.94 | 535.75±12.94 | 555.05±14.74 | 638.22±2.44 | 547.13±2.26 | 569.28±3.50 | 593.51±28.87 | 539.78±16.35 |
| 8 | hept-2-enal-D | 115.04±4.18 | 180.14±48.47 | 155.00±14.73 | 146.75±11.64 | 152.72±8.43 | 164.86±2.88 | 140.70±7.36 | 166.56±3.28 | 143.10±7.88 | 116.44±3.03 |
| 9 | methyl-5-hepten-2-one | 87.49±5.80 | 91.88±15.70 | 91.27±1.91 | 81.79±5.75 | 85.44±0.52 | 70.51±3.80 | 75.74±3.27 | 136.91±1.12 | 71.01±1.42 | 82.07±5.32 |
| 10 | oct-1-en-3-ol | 125.22±3.00 | 146.01±10.08 | 127.781.30± | 123.54±7.73 | 135.09±3.45 | 131.33±4.59 | 123.76±4.15 | 163.45±9.31 | 113.94±3.58 | 115.59±4.00 |
| 11 | Benzaldehyde | 98.22±2.85 | 128.07±4.40 | 112.77±1.99 | 120.17±2.62 | 100.64±2.75 | 104.28±1.59 | 110.34±5.52 | 132.13±4.42 | 118.60±3.73 | 123.47±7.32 |
| 12 | dihydro-2(3h)-furanone | 79.15±7.49 | 72.43±3.14 | 84.13±7.46 | 89.09±3.36 | 74.56±2.99 | 52.76±19.84 | 84.73±25.00 | 75.92±10.11 | 92.52±12.40 | 95.34±7.96 |
| 13 | 3 | 97.64±8.89 | 95.87±0.85 | 103.05±10.36 | 106.04±3.03 | 89.28±8.53 | 74.05±3.80 | 97.79±24.31 | 96.10±9.31 | 87.49±5.93 | 80.88±3.72 |

| | | | | | | | | | | | |
|---|---|---|---|---|---|---|---|---|---|---|---|
| 14 | Butyl acrylate | 344.72±5.38 | 403.77±59.95 | 412.07±3.59 | 355.48±13.06 | 338.93±3.93 | 500.53±5.18 | 364.17±4.59 | 354.61±8.99 | 331.34±10.12 | 301.51±8.79 |
| 15 | 2-heptanone | 1200.03±17.30 | 1552.66±239.22 | 1413.91±9.56 | 1429.11±30.72 | 1423.09±13.14 | 735.26±25.18 | 1260.73±26.26 | 1759.60±25.54 | 1016.40±43.13 | 1113.00±48.50 |
| 16 | n-Hexanol | 2486.73±47.22 | 2836.28±366.54 | 2602.75±19.54 | 2684.43±54.62 | 2750.98±37.32 | 1762.99±57.34 | 2502.42±24.16 | 2773.83±36.83 | 2237.37±70.90 | 2316.29±61.26 |
| 17 | hex-2-enal-M | 194.06±5.62 | 223.11±9.38 | 224.13±4.76 | 203.01±4.88 | 199.88±6.45 | 290.84±6.28 | 205.93±2.39 | 216.31±1.88 | 226.19±1.91 | 211.21±2.19 |
| 18 | hex-2-enal-D | 181.79±9.10 | 237.68±35.40 | 211.08±3.87 | 200.44±8.82 | 185.95±7.99 | 154.76±6.92 | 174.63±1.46 | 204.65±5.66 | 169.70±9.20 | 190.17±12.05 |
| 19 | 2-Butoxyethanol | 49.99±3.56 | 67.54±13.89 | 53.65±0.31 | 53.60±0.77 | 55.36±1.42 | 31.86±1.46 | 65.49±7.81 | 58.96±0.82 | 56.86±3.00 | 66.55±1.26 |
| 20 | 2-n-Butylfuran | 91.70±1.22 | 118.62±16.04 | 122.88±1.80 | 120.03±1.86 | 101.52±3.24 | 69.94±3.63 | 98.04±0.42 | 127.45±1.44 | 80.36±3.01 | 79.09±1.38 |
| 21 | 4 | 65.14±2.24 | 72.62±12.45 | 80.38±2.59 | 76.13±3.59 | 74.27±1.19 | 45.10±4.02 | 71.20±2.21 | 84.29±1.00 | 80.18±2.84 | 79.99±2.03 |
| 22 | Furfural | 21.64±0.50 | 18.56±1.56 | 17.09±0.34 | 17.92±0.86 | 19.73±1.02 | 14.42±0.22 | 17.34±0.47 | 15.35±1.21 | 21.04±0.38 | 20.05±1.81 |
| 23 | 5 | 30.31±1.90 | 34.46±5.25 | 30.34±1.75 | 39.33±4.14 | 32.09±1.57 | 41.15±0.48 | 29.54±1.05 | 30.81±0.34 | 29.59±3.81 | 30.93±3.87 |
| 24 | methyl 2-methylbutanoate | 34.07±2.23 | 39.32±2.75 | 40.56±2.72 | 39.01±0.92 | 39.28±0.95 | 44.06±0.40 | 44.22±1.40 | 53.09±2.81 | 41.98±1.83 | 41.45±1.41 |
| 25 | Hexanal | 2910.64±8.26 | 3360.54±78.27 | 3480.94±14.64 | 3240.13±28.83 | 3202.66±24.37 | 3486.52±24.22 | 3112.29±17.74 | 3664.98±13.43 | 3120.43±42.91 | 2995.09±12.53 |
| 26 | pentan-1-ol | 2049.75±22.12 | 2357.41±176.96 | 2221.06±12.37 | 2184.33±22.58 | 2215.88±17.40 | 1169.25±24.45 | 2096.52±34.85 | 2318.69±15.03 | 1968.03±37.34 | 2218.01±23.86 |
| 27 | 6 | 28.55±2.58 | 25.50±3.19 | 25.92±0.79 | 25.92±0.59 | 29.44±2.78 | 30.41±5.69 | 34.01±1.44 | 34.33±3.41 | 30.84±1.40 | 28.84±1.13 |
| 28 | Butyl acetate | 45.96±1.94 | 55.62±4.77 | 56.46±2.09 | 50.21±2.52 | 52.44±1.49 | 31.17±2.47 | 40.23±0.69 | 62.37±2.36 | 38.78±4.57 | 48.87±1.76 |
| 29 | 7 | 90.98±5.46 | 99.75±16.13 | 91.69±3.69 | 92.83±3.19 | 92.30±6.01 | 121.07±4.75 | 90.82±5.77 | 99.32±3.24 | 94.22±5.86 | 85.25±0.47 |
| 30 | 8 | 85.05±2.46 | 80.87±9.46 | 82.70±3.86 | 80.99±4.73 | 90.82±4.28 | 56.36±2.93 | 73.04±5.33 | 83.38±3.47 | 78.88±6.52 | 93.99±8.21 |
| 31 | 2-pentenal-M | 28.70±1.75 | 28.31±3.61 | 32.86±2.14 | 31.04±2.45 | 35.53±0.77 | 34.18±1.03 | 30.48±1.56 | 33.58±1.11 | 38.91±2.13 | 36.24±1.37 |

| | | | | | | | | | | | |
|---|---|---|---|---|---|---|---|---|---|---|---|
| 32 | 9 | 45.87±1.79 | 52.93±6.20 | 59.70±1.91 | 55.43±1.63 | 58.62±0.71 | 52.83±1.54 | 54.21±1.32 | 70.50±1.02 | 57.98±2.31 | 54.16±1.32 |
| 33 | 2-methylbutan-1-ol | 220.04±2.92 | 335.42±41.10 | 310.43±2.28 | 339.55±8.91 | 285.10±2.84 | 252.32±1.49 | 294.55±1.92 | 399.60±3.57 | 289.45±10.72 | 250.19±0.19 |
| 34 | 3-methylbutan-1-ol | 403.76±1.91 | 494.63±45.03 | 407.98±6.10 | 511.92±14.48 | 379.00±6.71 | 441.14±9.64 | 435.77±0.86 | 535.70±3.29 | 442.39±12.10 | 384.76±8.30 |
| 35 | n-Propyl acetate-D | 58.38±2.19 | 87.27±23.32 | 74.72±1.93 | 80.44±6.17 | 82.80±1.22 | 28.55±2.73 | 94.52±3.33 | 87.16±2.13 | 56.99±4.76 | 108.57±4.83 |
| 36 | Pentanal | 190.96±4.04 | 341.69±10.88 | 385.30±2.85 | 299.98±6.58 | 283.37±6.46 | 173.11±14.05 | 266.72±0.88 | 470.30±10.77 | 252.83±12.44 | 300.64±6.34 |
| 37 | 1-Propanethiol | 349.19±6.09 | 359.72±33.56 | 276.79±2.47 | 298.70±1.36 | 270.70±9.04 | 414.10±26.67 | 338.67±5.13 | 277.02±9.84 | 361.65±19.17 | 337.78±5.94 |
| 38 | Ethyl Acetate | 2324.06±46.11 | 2183.85±244.17 | 2032.32±18.38 | 2379.86±44.81 | 2205.16±12.08 | 426.72±9.73 | 2477.41±25.24 | 1542.86±4.76 | 1864.39±36.05 | 2457.48±47.61 |
| 39 | 2-Butanone | 658.11±7.48 | 1083.13±155.95 | 902.56±11.07 | 951.38±4.35 | 721.47±9.40 | 812.30±7.51 | 756.90±6.16 | 1312.52±5.48 | 763.49±12.88 | 838.01±6.76 |
| 40 | acetone | 3924.75±14.17 | 4465.28±37.60 | 4712.9810.17 | 4451.25±61.47 | 4623.36±29.87 | 4248.68±49.80 | 4186.77±54.83 | 4404.24±29.56 | 4194.88±37.89 | 4251.66±43.62 |
| 41 | ethanol | 1171.04±13.60 | 1128.67±59.43 | 1136.64±9.57 | 1152.96±45.51 | 1142.47±40.79 | 1059.33±45.70 | 1155.65±46.36 | 1150.82±41.61 | 1050.90±82.26 | 1093.19±7.92 |
| 42 | 10 | 1373.72±18.50 | 1198.72±34.48 | 1097.73±2.43 | 1170.51±24.20 | 1206.36±25.22 | 721.58±17.93 | 1225.41±40.51 | 1037.69±29.00 | 1197.12±97.26 | 1141.87±79.92 |
| 43 | 2-methylbutanal | 164. 71±5.57 | 143.77±24.95 | 151.49±2.67 | 146.41±3.05 | 156.86±4.51 | 185.43±0.81 | 161.39±6.87 | 138.10±4.39 | 165.27±5.84 | 146.78±8.98 |

| | | | | | | | | | | | |
|---|---|---|---|---|---|---|---|---|---|---|---|
| 44 | 3-methylbutanal | 307.76±4.04 | 309.80±5.91 | 308.49±5.02 | 308.41±8.17 | 312.94±8.98 | 372.08±7.96 | 337.28±15.20 | 297.81±20.14 | 329.12±30.96 | 326.29±17.40 |
| 45 | 2-Pentanone | 78.87±3.13 | 145.85±21.30 | 125.72±1.28 | 142.30±3.99 | 117.16±2.85 | 92.94±3.86 | 121.52±2.57 | 211.02±4.56 | 102.96±3.44 | 112.56±1.73 |
| 46 | butanal | 137.45±4.98 | 165.11±21.59 | 142.84±6.10 | 153.64±7.93 | 128.56±10.68 | 183.15±10.48 | 167.04±8.03 | 168.77±11.50 | 172.95±15.49 | 168.36±5.65 |
| 47 | 11 | 111.32±2.25 | 156.96±27.35 | 165.17±2.00 | 144.27±5.82 | 147.34±4.36 | 98.01±3.03 | 123.77±3.08 | 160.11±3.28 | 140.40±3.15 | 130.74±2.28 |
| 48 | 2-pentenal-D | 75.26±1.48 | 83.18±12.17 | 82.55±5.00 | 68.90±0.79 | 72.34±1.20 | 63.95±1.24 | 62.83±1.81 | 60.37±2.26 | 75.61±1.39 | 72.37±3.39 |
| 49 | 12 | 232.13±2.06 | 238.18±23.75 | 257.80±3.16 | 260.81±9.02 | 265.73±5.34 | 219.93±9.98 | 260.55±9.23 | 288.65±9.38 | 239.52±8.83 | 252.11±5.60 |
| 50 | n-Propyl acetate-M | 105.71±3.60 | 107.56±6.11 | 96.97±4.26 | 107.51±1.96 | 104.16±2.40 | 77.83±3.71 | 113.40±5.78 | 98.73±1.12 | 99.67±3.63 | 112.86±0.18 |

[a] The signal intensities of the volatile compounds tentatively identified in 20 varieties of foxtail millet by HS-GC-IMS were present as mean±standard deviation.

**Table S2.**

Contents (mean±standard deviation) of the volatile compounds tentatively identified in 20 varieties of foxtail millet by HS-SPME/GC-MS

| No. | Componds | Contents (μg/g) | | | | | | | | | |
|---|---|---|---|---|---|---|---|---|---|---|---|
| | | 77-322 | Jingu 21 | Jingu 26 | Jingu 28 | Jingu 34① | Jingu 34② | Jingu 34③ | Jingu 41 | Jingu 42 | Jingu 46 |
| 1 | Hexanal | 5.86±0.21 | 5.56±0.01 | 5.49±0.16 | 6.44±0.09 | 6.17±0.08 | 5.57±0.11 | 5.96±0.05 | 6.18±0.04 | 6.17±0.07 | 6.31±0.02 |
| 2 | Benzaldehyde | 0.16±0.02 | 0.21±0.02 | 0.22±0.02 | 0.20±0.01 | 0.18±0.01 | 0.19±0.01 | 0.18±0.01 | 0.17±0.01 | 0.16±0.01 | 0.16±0.01 |
| 3 | Heptanal | 5.44±0.10 | 3.44±0.02 | 5.63±0.06 | 5.54±0.04 | 5.36±0.04 | 3.69±0.02 | 5.60±0.20 | 5.41±0.09 | 4.03±0.09 | 4.58±0.16 |
| 4 | Octanal | 0.33±0.02 | 0.25±0.01 | 0.52±0.02 | 0.51±0.02 | 0.37±0.01 | 0.28±0.01 | 0.37±0.01 | 0.43±0.01 | 0.27±0.03 | 0.28±0.01 |
| 5 | Nonanal | 1.11±0.07 | 1.26±0.04 | 1.34±0.14 | 1.17±0.04 | 1.56±0.06 | 1.42±0.11 | 1.59±0.04 | 1.18±0.06 | 0.79±0.02 | 0.58±0.02 |
| 6 | Decanal | 0.36±0.03 | ND | 0.13±0.02 | 0.12±0.03 | 0.08±0.01 | ND | 0.11±0.01 | 0.10±0.03 | 0.16±0.01 | 0.13±0.02 |
| 7 | 2-Heptanone | 2.82±0.03 | 1.55±0.04 | 3.09±0.04 | 3.23±0.05 | 3.00±0.02 | 1.79±0.02 | 2.73±0.04 | 3.12±0.03 | 1.80±0.02 | 2.11±0.05 |
| 8 | Acetophenone | 4.87±0.18 | 4.57±0.15 | 4.65±0.05 | 5.44±0.07 | 5.22±0.05 | 4.71±0.02 | 4.87±0.02 | 5.10±0.15 | 4.89±0.04 | 5.10±0.04 |
| 9 | 3-Octen-2-one | 0.23±0.01 | 0.16±0.01 | 0.36±0.01 | 0.27±0.01 | 0.23±0.01 | 0.17±0.01 | 0.23±0.01 | 0.28±0.01 | 0.16±0.01 | 0.18±0.01 |
| 10 | 1-Pentanol | 4.60±0.05 | 3.55±0.02 | 4.64±0.10 | 4.60±0.03 | 4.75±0.04 | 3.54±0.01 | 4.60±0.09 | 4.59±0.07 | 3.14±0.01 | 3.61±0.03 |
| 11 | 1-Hexanol | 1.11±0.02 | 1.00±0.01 | 1.13±0.02 | 1.25±0.02 | 1.10±0.01 | 0.93±0.01 | 1.06±0.03 | 1.07±0.02 | 0.77±0.01 | 0.78±0.03 |
| 12 | 1-Octen-3-ol | 0.29±0.04 | 0.23±0.01 | 0.41±0.01 | 0.33±0.01 | 0.30±0.01 | 0.24±0.01 | 0.30±0.01 | 0.34±0.02 | 0.19±0.01 | 0.21±0.01 |
| 13 | Tetradecane | 0.15±0.01 | 0.14±0.01 | 0.17±0.01 | 0.16±0.01 | 0.18±0.01 | 0.16±0.01 | 0.16±0.01 | 0.18±0.01 | 0.11±0.01 | 0.11±0.01 |
| 14 | Pentadecane | 0.08±0.02 | 0.09±0.01 | 0.10±0.01 | 0.09±0.01 | 0.09±0.01 | 0.09±0.01 | 0.07±0.01 | 0.11±0.02 | 0.05±0.01 | ND |
| 15 | Hexadecane | 0.12±0.01 | 0.13±0.01 | 0.11±0.01 | 0.13±0.01 | 0.12±0.01 | 0.17±0.01 | 0.11±0.01 | 0.12±0.01 | 0.08±0.01 | 0.09±0.01 |
| 16 | Nonadecane | 0.45±0.02 | 0.54±0.02 | 0.38±0.01 | 0.51±0.02 | 0.46±0.01 | 0.58±0.01 | 0.45±0.01 | 0.39±0.02 | 0.35±0.01 | 0.38±0.01 |
| 17 | Benzothiazole | 0.16±0.02 | 0.21±0.01 | 0.22±0.01 | 0.20±0.01 | 0.18±0.01 | 0.19±0.01 | 0.15±0.01 | 0.17±0.01 | 0.17±0.01 | 0.16±0.01 |
| 18 | Benzene, 1,3-dimethyl- | 0.16±0.01 | 0.16±0.01 | 0.17±0.01 | 0.16±0.01 | 0.22±0.01 | 0.17±0.01 | 0.21±0.02 | 0.18±0.01 | 0.13±0.01 | 0.12±0.01 |

| No. | Componds | Contents (μg/g) | | | | | | | | | |
|---|---|---|---|---|---|---|---|---|---|---|---|
| | | Jingu 48 | Jingu 53 | Jingu 54 | Jingu 55 | Jingu 58 | Jingu 59 | Jingu 60 | Jingu 62 | Changnong35 | Changsheng07 |
| 1 | Hexanal | 6.13±0.02 | 6.98±0.06 | 7.36±0.02 | 6.96±0.10 | 7.22±0.05 | 6.64±0.08 | 6.54±0.09 | 6.88±0.05 | 6.55±0.06 | 6.64±0.07 |
| 2 | Benzaldehyde | 0.15±0.01 | 0.20±0.01 | 0.18±0.01 | 0.19±0.01 | 0.16±0.01 | 0.16±0.01 | 0.17±0.01 | 0.21±0.01 | 0.19±0.01 | 0.19±0.01 |
| 3 | Heptanal | 3.89±0.07 | 4.10±0.09 | 4.07±0.03 | 4.19±0.09 | 4.30±0.06 | 2.75±0.09 | 3.91±0.04 | 4.33±0.06 | 3.50±0.11 | 3.62±0.10 |
| 4 | Octanal | 0.27±0.01 | 0.36±0.04 | 0.33±0.01 | 0.33±0.01 | 0.28±0.01 | 0.25±0.01 | 0.26±0.01 | 0.31±0.01 | 0.24±0.02 | 0.22±0.02 |
| 5 | Nonanal | 0.77±0.01 | 0.79±0.06 | 0.82±0.02 | 1.00±0.02 | 0.67±0.04 | 1.52±0.03 | 0.72±0.06 | 0.71±0.04 | 0.72±0.02 | 0.73±0.03 |
| 6 | Decanal | 0.13±0.04 | 0.06±0.03 | 0.06±0.01 | 0.07±0.01 | 0.14±0.02 | 0.05±0.01 | 0.11±0.04 | 0.15±0.02 | 0.10±0.03 | 0.11±0.05 |
| 7 | 2-Heptanone | 1.88±0.03 | 2.23±0.03 | 2.21±0.01 | 2.23±0.05 | 2.22±0.02 | 1.15±0.04 | 1.97±0.04 | 1.75±0.04 | 1.59±0.07 | 1.74±0.08 |
| 8 | Acetophenone | 4.55±0.01 | 5.32±0.04 | 5.44±0.02 | 5.06±0.05 | 5.00±0.04 | 5.45±0.04 | 4.86±0.03 | 4.73±0.02 | 4.88±0.07 | 4.68±0.02 |
| 9 | 3-Octen-2-one | 0.17±0.01 | 0.22±0.01 | 0.26±0.01 | 0.23±0.01 | 0.23±0.01 | 0.15±0.01 | 0.19±0.01 | 0.25±0.01 | 0.22±0.01 | 0.20±0.01 |
| 10 | 1-Pentanol | 3.20±0.03 | 3.53±0.05 | 3.47±0.02 | 3.41±0.04 | 3.46±0.03 | 1.83±0.04 | 3.28±0.05 | 3.62±0.02 | 3.08±0.06 | 3.47±0.04 |
| 11 | 1-Hexanol | 0.78±0.01 | 0.87±0.03 | 0.87±0.02 | 0.84±0.02 | 0.87±0.02 | 1.00±0.01 | 0.85±0.01 | 0.89±0.01 | 0.93±0.05 | 0.84±0.03 |
| 12 | 1-Octen-3-ol | 0.20±0.01 | 0.23±0.02 | 0.20±0.01 | 0.19±0.01 | 0.21±0.01 | 0.21±0.01 | 0.19±0.01 | 0.16±0.01 | 0.18±0.01 | 0.18±0.01 |
| 13 | Tetradecane | 0.10±0.01 | 0.11±0.02 | 0.13±0.01 | 0.12±0.01 | 0.12±0.01 | ND | 0.11±0.01 | 0.13±0.01 | 0.13±0.01 | 0.12±0.01 |
| 14 | Pentadecane | 0.04±0.01 | ND | 0.04±0.01 | 0.04±0.01 | 0.05±0.01 | 0.05±0.01 | 0.05±0.01 | 0.05±0.01 | 0.05±0.01 | 0.05±0.01 |
| 15 | Hexadecane | ND | 0.08±0.01 | 0.09±0.01 | 0.09±0.01 | 0.09±0.01 | 0.08±0.01 | 0.08±0.01 | 0.11±0.01 | 0.09±0.01 | 0.08±0.01 |
| 16 | Nonadecane | 0.36±0.01 | 0.39±0.01 | 0.40±0.01 | 0.41±0.01 | 0.42±0.01 | 0.34±0.02 | 0.41±0.01 | 0.45±0.01 | 0.37±0.01 | 0.39±0.01 |
| 17 | Benzothiazole | 0.15±0.01 | 0.20±0.01 | 0.18±0.01 | 0.19±0.01 | 0.16±0.01 | 0.16±0.01 | 0.17±0.01 | 0.21±0.01 | 0.19±0.01 | 0.19±0.01 |
| 18 | Benzene, 1,3-dimethyl- | 0.14±0.01 | 0.14±0.01 | 0.14±0.01 | 0.15±0.01 | 0.14±0.01 | 0.19±0.01 | 0.14±0.01 | 0.16±0.01 | 0.15±0.01 | 0.13±0.01 |

ND: Not detected.
